# Artificial intelligence for imaging-based COVID-19 detection: Systematic review comparing added value of AI versus human readers

**DOI:** 10.1016/j.ejrad.2021.110028

**Published:** 2021-12

**Authors:** Christine Kriza, Valeria Amenta, Alexandre Zenié, Dimitris Panidis, Hubert Chassaigne, Patricia Urbán, Uwe Holzwarth, Aisha Vanessa Sauer, Vittorio Reina, Claudius Benedict Griesinger

**Affiliations:** European Commission, Joint Research Centre (JRC), Via E. Fermi 2749 (TP 281) Ispra, Lombardy, Italy

## Abstract

**Purpose:**

A growing number of studies have examined whether Artificial Intelligence (AI) systems can support imaging-based diagnosis of COVID-19-caused pneumonia, including both gains in diagnostic performance and speed. However, what is currently missing is a combined appreciation of studies comparing human readers and AI.

**Methods:**

We followed PRISMA-DTA guidelines for our systematic review, searching EMBASE, PUBMED and Scopus databases. To gain insights into the potential value of AI methods, we focused on studies comparing the performance of human readers versus AI models or versus AI-supported human readings.

**Results:**

Our search identified 1270 studies, of which 12 fulfilled specific selection criteria. Concerning diagnostic performance, in testing datasets reported sensitivity was 42–100% (human readers, n = 9 studies), 60–95% (AI systems, n = 10) and 81–98% (AI-supported readers, n = 3), whilst reported specificity was 26–100% (human readers, n = 8), 61–96% (AI systems, n = 10) and 78–99% (AI-supported readings, n = 2). One study highlighted the potential of AI-supported readings for the assessment of lung lesion burden changes, whilst two studies indicated potential time savings for detection with AI.

**Conclusions:**

Our review indicates that AI systems or AI-supported human readings show less performance variability (interquartile range) in general, and may support the differentiation of COVID-19 pneumonia from other forms of pneumonia when used in high-prevalence and symptomatic populations. However, inconsistencies related to study design, reporting of data, areas of risk of bias, as well as limitations of statistical analyses complicate clear conclusions. We therefore support efforts for developing critical elements of study design when assessing the value of AI for diagnostic imaging.

## Highlights

• There may be an added value of AI model supported imaging-based COVID-19 detection.
• Studies reported comparable or better performance of AI or AI-supported readings.
• There was lower variability of diagnostic performance for AI than for human readers.
• Our systematic review shows heterogeneity of data characteristics and risks of bias.
• There is a variety of applied methodologies and statistical analysis limitations.

## 1 Introduction

The field of medical imaging has seen rapid progress in the last years for the application of artificial intelligence (AI) methodologies, especially machine learning (ML) and deep learning (DL) [1]. AI systems have progressed in image-recognition tasks relevant to disease diagnosis and detection, thus mimicking expert data interpretation capacities [2]. Recent studies have shown that AI-supported imaging technologies for specific diseases have a diagnostic performance comparable to medical experts [3].

According to the WHO, there have been over 247 million confirmed cases of COVID-19 globally as of November 3, 2021 and the pandemic has claimed over 5 million deaths worldwide [4]. COVID-19 diagnosis is largely based on reverse transcriptase-polymerase chain reaction (RT-PCR) testing, but problems related to test kit availability, test reliability, and test turnaround time have persisted in many countries. Additional fast, low-cost, and easily scalable tools for triaging and detecting COVID-19 suspected patients are crucial [5]. The COVID-19 pandemic has provided considerable momentum to this research area, with high expectations from the clinical community, which warrants an overview assessing the current evidence regarding the potential of AI methods to support the accurate, and fast detection of COVID-19 pneumonia. However, the accurate differentiation between COVID-19 and pneumonia of other origins remains challenging, due to subtle radiologic differences, especially in asymptomatic patients and those with early onset of symptoms [5].

We performed a systematic review of peer-reviewed publications that used AI for the evaluation of lung imaging to support the detection of COVID-19, and compared findings between a selected AI system and human readers, or AI-supported readings versus human readers alone, to obtain a comprehensive view of the current status of published evidence. To our knowledge, this is the first published systematic review to date on this focused topic. Due to the large heterogeneity in the reporting of relevant results, the information was not amenable to quantitative data grouping or *meta*-analysis. We therefore focused our study on the following aims: (a) firstly, to compare the evidence from published studies that used AI methodologies for supporting the detection of COVID-19 in lung imaging and also included an element of comparison between the performance values of human readers versus AI models or human readers versus AI-supported readings, (b) secondly, to look into the consistency of the reporting of outcomes across different studies, and (c) finally, to assess the risk of bias in the published studies according to a standard appraisal tool.

## 2 Material and methods

### 2.1 Search strategy and selection criteria

Our systematic review was performed in accordance with the Preferred Reporting Items for Systematic Reviews and Meta-Analyses of Diagnostic Test Accuracy (PRISMA-DTA), including the related checklist [6], [7]. We used the systematic review methods outlined in the York Centre for Reviews and Dissemination’s guidance for undertaking reviews in healthcare, specifically focusing on the guidance for systematic reviews of clinical tests [8].

The relevant population included COVID-19 pneumonia positive patients versus those with other pneumonia symptoms that tested negative for COVID-19. The intervention or index test was imaging, which was evaluated by both AI and human readers. As a comparator or reference standard, a positive PCR test was most commonly used in current practice, but in some cases they were supported by radiologists’ findings and a clinical consensus. We were interested in the outcome measures related to the support of COVID-19 detection, comparing AI and human readers, i.e. sensitivity, specificity per patient, and related values, such as AUC (area under the Receiver Operator Characteristic -ROC- curve). We also explored additional outcomes, such as time required for diagnosis.

We only included peer-reviewed studies in our review. The included studies had to focus on supporting the detection of COVID-19 with a lung-imaging modality, and applying an AI methodology for the analysis of the imaging outcomes, including ML and DL. Furthermore, the studies had to include a comparison between human readers versus AI models or human readers versus AI-supported readings and report outcomes related to sensitivity and specificity at least, but ideally also further outcome measures. We excluded studies not focusing on lung imaging of COVID-19, for example those using ex-vivo imaging and pathology studies, and those focused on aspects related to segmentation, features extraction, treatment, survival and disease risk prediction (see appendix, table 1 for full list of selection criteria).

We conducted a systematic search of the literature including EMBASE, PubMed and Scopus databases for papers published from January 1, 2019 in the English language (see appendix, table 2 for search strings). The searches were performed on November 30, 2020. Additional papers were identified through automated database notifications of new publications according to our search terms until January 31, 2021. The reference lists from all included papers were checked to identify and include any other potential studies.

Two reviewers independently performed a screening of the citations by title and abstract, with discrepancies resolved by consensus. The citations identified in the systematic search were uploaded to EndNote reference manager and duplicates were automatically deleted.

### 2.2 Quality assessment

Two reviewers acquired the full-text versions of the included papers and independently assessed their methodological quality using the QUADAS-2 tool, which has been adapted to the systematic review objectives (see overview of signalling questions in appendix, table 3). Any discrepancies were resolved through discussion between the reviewers. QUADAS-2 is an appraisal tool recommended for the use in systematic reviews to evaluate the risk of bias in diagnostic accuracy studies [9]. QUADAS-2 consists of the following four domains: patient selection, index test, reference standard and flow and timing. We used an additional domain of data management and assessed all five domains for risk of bias using relevant signalling questions. While QUADAS-2 is not intended to generate an overall score, the tool highlights a high, medium or unclear risk of bias according to the domains assessed. We excluded studies, with a high risk of bias in at least two domains, in the final analysis of our systematic review. In addition, one reviewer evaluated the methodologies applied for statistical analysis on all available datasets, where appropriate performance measurements were reported in the included papers.

### 2.3 Data analysis

Two reviewers extracted the following information on study characteristics for each paper, including: study category, imaging modality, country where the imaging took place, experience of human readers, selection criteria in the original research studies, reference standards used and information on blinding, as well as sample sizes for different datasets and validation type (Table 1a). In addition, information on the AI method and the data source, together with the data acquisition time period were extracted (Table 1b). Along with the original study authors’ original conclusions, the following outcome data were extracted per-patient where applicable: values for sensitivity, specificity, accuracy, positive predictive value, negative predictive value and AUC. Where indicated, other values were extracted for speeds of reading and potential time savings (Table 2).

**Table 1a:** Study characteristics.

	Study Category	Imaging modality and country where imaging took place	Experience of human readers	Reference standard and information on blinding	Total dataset patient characteristics and sample size (where indicated or calculated)	Training dataset patient characteristics and sample size (where indicated or calculated)	Validation dataset patient characteristics and sample size (where indicated or calculated)	Validation type	Testing dataset patient characteristics and sample size (where indicated or calculated)
Lessmann (26)	Comparison of AI model with 8 human readers	CT, Netherlands	8 readers: 4 readers with<5 y, 4 readers up to 27 y	Reference standard not sufficiently detailed, AI model was compared with human readers and to clinical assessment of the patients, including RT-PCR testing Information on reader blinding provided	843	476, mean age 60 (+/-16), male 56%	Not stated	Claimed external validation, but without providing performance measurements	Internal test set: 104/105, mean age 62 (+/-16), male 58%; External test set: 262, mean age 64 (+/-16), male 54%
Zhang Ran (18)	Comparison of AI model with 3 human readers	CXR, USA	3 readers, all experienced thoracic radiologists, with over 9, 14, and 34 y of experience	Reader consensus between three radiologists was used as reference standard Information on reader blinding provided	5 208, out of which 2 060 with COVID-pneumonia (mean age 62 years, +/- 16, 51% male) and 3 148 with non-COVID-pneumonia (mean age 64 years, +/-18, 50·1% male)	Training and validation combined: 3015	Training and validation combined: 3 015	Claimed validation, but without providing performance measurements	2193
Wang (22)	Comparison of AI model with a radiologist panel of 2 human readers; focus on the identification of lesion burden increase on pairs of CT scans obtained from 100 patients with COVID-19·	CT, China	n/a	Data for both RT-PCR and radiological findings as reference standards provided Information on reader blinding provided	5 206	1 958	Independent internal validation: 639, median 55 years (38–66), male 48%; external validation: 2120, median 43 years (31–56), male 51%	Independent internal and external validation	489
Harrison (16)	Comparison of 6 human readers with and without assistance of AI model; performance measurements are also reported for the AI model only	CT, USA and China	6 readers, 3 radiologists with 10 y experience, 3 radiologists with 20 y of chest CT experience	Reader consensus was used as reference standard Information on reader blinding provided	1 186 (provided separately for COVID-19 and non COVID-19 group, calculated 55% male, 45% female on the total number)· Mean age provided separately per COVID-19 group (46 ± 16) and non COVID-19 group (62 ± 19)	830	237	Internal validation	119
Mei (28)	Comparison of AI model with 2 human readers	CT, China	2 readers, one fellowship-trained thoracic radiologist with 10 y experience and one thoracic radiology fellow	RT-PCR was used as reference standard Information on reader blinding provided	905 (mean age 40·7 years, +/- 16·5 years), 54% males	Training: 534, tuning: 92	Not stated	Not stated	279
Murphy (20)	Comparison of AI model with 6 human readers	CXR, Netherlands	6 readers, all chest radiologists with experience ranging from 5 y up to more than 30 y	RT-PCR was used as reference standard Information on reader blinding provided	25 146	23 138, mean age 47·8+/-17·0 years, 57·6% male, 43·2% female, 0·2% unknown	1 540, mean age 47·4+/-17·1 years, 56·7% male	Claimed validation, but without providing performance measurements	468, mean age 67·3+/-14·4 years, 55·8% male
Wehbe (17)	Comparison of AI model with 5 human readers	CXR, USA	5 readers, with 4 board-certified thoracic radiologists with experience ranging from 1 to 8 y and one board-certified diagnostic radiologist with 38 y of post-training experience	Results for both options with RT-PCR and using consensus interpretation as reference standard indicated Information on reader blinding provided	5 853 (mean age 58 +/-19 years, 47% male)	3 931	1 100	Internal validation	866
Zhou Min (27)	Comparison of AI models with 10 human readers in two groups (specialist-level and resident-level)	CT, China	10 radiologists from two groups (group 1, 5 specialist-level radiologists with more than 15 y of experience; group 2, 5 resident radiologists with 3–5 y of experience) in thoracic imaging	Reference standard not sufficiently detailed Information on reader blinding provided	449 (gender provided separately for NCP and IP groups, calculated 57% male, 43% female on the overall number)· Mean age provided separately for influenza pneumonia group (IP) and novel coronavirus pneumonia group (NCP)	275	33 independent internal + 107 external (calculated 60% male, 40% female on the external set)	Independent internal validation and external validation	34
Castiglioni (21)	Comparison of AI model with 2 human readers	CXR, Italy	2 readers, 1 radiologist with 15 y of experience in chest imaging at Centre 1 and a general radiologist with 6 y of experience at Centre 2	RT-PCR was used as reference standard Information on reader blinding provided	610	Training & validation: 500; Mean age provided separately for COVID-19 and non COVID-19 group and for Centre 1 and Centre 2, gender provided separately for centre 1 (55% male) and 2 (65% male), calculated as 60% male and 40% female on the tot of 500	Training & validation: 500 Mean age provided separately for COVID-19 and non COVID-19 group and for Centre 1 and Centre 2, gender provided separately for centre 1 (55% male) and 2 (65% male), calculated as 60% male and 40% female on the tot of 500	Claimed validation, but without providing performance measurements	110
Wang Hongmei (23)	Comparison of 3 human readers with and without assistance of AI model, performance measurements are also reported for the AI model only	CT, China and USA	3 readers with over 5 y of experience	Reference standard not sufficiently detailed Information on reader blinding provided	216 (59·3% male, age medians available for patients selected from three different hospitals, total age median 44 years)	148	18 internal validation, 32 external validation	Independent internal and external validation datasets	18
Chiu (5)	Comparison of AI model with 3 human readers	CXR, Hong Kong	3 readers, all board-certified radiologists, each with > 10 y experience)	Reference standard not sufficiently detailed, RT-PCR as well as radiologic reference standard mentioned Information on reader blinding provided	762	514, median age 65 (44–82), 52·3 % male, 47·7 % female	248, median age 61 (39–79), 51% male, 49% female	Prospectively collected independent validation set representing a real-world mix of patients screened for COVID-19	not stated
Yang Yanhong (24)	Comparison of 3 human readers with and without assistance of AI model, performance measurements are also reported for the AI model only	CT, China	3 radiologists with over 20 y, nearly 20 y and 5∼10 y of chest CT experience	Final diagnosis decision from the hospital, also based on radiologists' readings, was used as reference standard Information on reader blinding provided	694	509 (ages available for subgroups, average age 49 years, 65·8% male)	Not stated	Claimed validation, but without providing performance measurements	185 (ages available for subgroups, average age 47, 61·6% male)

**Legend:** AI: Artificial Intelligence, CT: Computed Tomography, CXR: Chest X-Ray, RT-PCR: Reverse transcription polymerase chain reaction, SARS-CoV-2: Severe Acute Respiratory Syndrome Coronavirus-2, y: years.

**Table 1b:** AI methods and data sources overview.

	AI method	Data source and data acquisition time period for model input	Inclusion/ Exclusion criteria in original research study
Lessmann (26)	Three successively applied deep learning algorithms were used for (1) pulmonary lobe segmentation and labeling, (2) CT severity score prediction and (3) CO-RADS score prediction.	Patients were included from an academic center and a large teaching hospital in the Netherlands in March and April of 2020 who met inclusion criteria.	**Inclusion criteria**: symptoms of lower respiratory tract infection including cough, clinically relevant dyspnoea requiring hospital admission and fever with anosmia; **exclusion criteria**: no CT severity scores in radiology report, COVID-19 prior to imaging, or missing RT-PCR test results RT-PCR was used to confirm COVID-19 patients
Zhang Ran (18)	The deep neural network CV19-Net was used. The CV19-Net used in this work is an ensemble of 20 individually trained deep neural networks.	Data from hospital chain in US, Henry Ford Health System, including 5 hospitals and more than 30 clinics (February 1 to May 30, 2020).	**Inclusion criteria for non-COVID-19 pneumonia group:** patients that underwent frontal view CXR, had pneumonia diagnosis, and imaging was performed in specified time period; **inclusion criteria for COVID-19 group:** patients that underwent frontal view CXR, with a RT-PCR positive test for SARS-CoV-2 with a diagnosis of pneumonia; **exclusion criteria:** patients under the age of 18 and those where CXR was performed more than 5 days prior or 14 days after RT-PCR confirmation· RT-PCR was used to confirm COVID-19 patients
Wang (22)	A U-Net-based deep learning model was used to segment lung opacities on chest CT, alert cases with COVID-19 imaging manifestations and lesion burden changes.	CT images were obtained from Tongji Hospital in Wuhan, China (Feb 1 to March 3, 2020). CT images and radiological reports were obtained from three fever clinics (Tianyou Hospital in Wuhan, China, Xianning Central Hospital in Xianning, China and The Second Xiangya Hospital in Changsha, China).	**Inclusion criteria COVID-19 group:** positive diagnosis confirmed by RT-PCR; pairs of CT scans available, patients older than 14 years; **inclusion criteria non-COVID-19 group:** double negative RT-PCR test results, with or without positive CT findings and scanning available, patients older than 14 years RT-PCR was used to confirm COVID-19 patients
Harrison (16)	To classify COVID-19 versus other pneumonia types for each patient, abnormal CT slices were used as input into the EfficientNet B4 deep neural network architecture after lung segmentation, followed by a two-layer fully connected neural network to pool slices together.	Data from 9 hospitals in Hunan Providence, China (January 6 to April 1, 2020) and the Rhode Island Hospital was used.	**Inclusion criteria for pneumonia group**: CT scans with report impression containing the word pneumonia; **exclusion criteria**: no chest CT scan, no clear signs of pneumonia or findings suggestive of another diagnosis on chest CT scan;· **inclusion criteria for COVID-19 group**: patients with positive diagnosis by RT-PCR and available CT; **exclusion criteria**: no abnormal findings on chest CT scans RT-PCR was used to confirm COVID-19 patients
Mei (28)	First, a deep convolutional neural network was developed to learn the imaging characteristics of patients with COVID-19 on the initial CT scan. Then a support vector machine, random forest and multilayer perceptron classifiers were used to classify patients with COVID-19 according to clinical information. A neural network model was created combining radiological data and clinical information to predict COVID-19 status.	A dataset of the presenting patients was acquired from 18 medical centers in 13 provinces in China (January 17 to March 3, 2020).	**Inclusion criteria** COVID-19 exposure, fever, RT-PCR test, chest CT scan RT-PCR was used to confirm COVID-19 patients
Murphy (20)	CAD4COVID-XRay was used, which is a deep learning–based AI system used to detect COVID-19 characteristics on frontal chest radiographs. CAD4COVID-Xray is based on CAD4TB version 6 software, which is a commercial deep learning system for the detection of tuberculosis on chest radiographs.	A publicly available CXR dataset (Radiological Society of North America pneumonia detection challenge) was used for retraining. The test set was selected from Jeroen Bosch Hospital in the Netherlands (March 4 and April 6, 2020). For fine-tuning the system, an additional training set was acquired from Bernhoven Hospital in the Netherlands. These were combined with images from other institutes and public sources, including from the Radboud University Medical Centre.	**Inclusion criteria** Individuals suspected of having COVID-19 who presented to the emergency department with respiratory symptoms; all patients underwent laboratory measurements, chest radiographic imaging, and RT-PCR testing RT-PCR was used to confirm COVID-19 patients
Wehbe (17)	DeepCOVID-XR was used, which is a weighted ensemble of deep neural networks.	The study sample included consecutive patients from over 20 sites (including hospitals, standalone emergency departments, and urgent care facilities) across the Northwestern Memorial Health Care (NMHC) System in the United States (February to April 2020).	**Inclusion criteria** Patients included adults >=18 years of age with either 1) a documented RT-PCR test result for SARS-CoV-2 (whether positive or negative), 2) a diagnosis of COVID-19 by International Classification of Diseases (ICD-10) code, or 3) a COVID-19 ‘definitive positive’ flag in the electronic health record ; COVID-19 positivity was defined as 1) any single positive RT-PCR result during the associated clinical encounter, 2) a diagnosis of COVID-19 by ICD-10 code, or 3) COVID-19 “definitive positive” flag in the electronic health record RT-PCR was used to confirm COVID-19 patients
Zhou Min (27)	A Deep learning model (the Trinary scheme) was used to discriminate influenza pneumonia lesions from novel coronavirus pneumonia lesions.	Data from 11 hospitals in China was used that met patient selection criteria. This included data from 4 novel coronavirus pneumonia designated hospitals (January 11 to February 23, 2020) and 5 hospitals for influenza pneumonia patients (May 2015 to February 2020). The external validation set included patient data from 8 hospitals.	**Inclusion criteria for COVID-19 pneumonia group:** confirmed positive RT-PCR and underwent CT scans within 4 days, **exclusion criteria** - negative pneumonia findings on CT; **inclusion criteria for influenza pneumonia group:** confirmed RT-PCR and underwent CT scans within four days, **exclusion criteria** - negative pneumonia findings on CT RT-PCR was used to confirm COVID-19 patients
Castiglioni (21)	A Deep learning classifier was used for the diagnosis of COVID-19. An ensemble of ten convolutional neural networks was then used for training and validation.	The training and validation set was composed of CXRs from the Hospital San Gerardo, Monza and the IRCCS Policlinico San Donato in Italy (March 1 to March 13, 2020).	**Inclusion criteria**: patients with clinical suspicion of COVID-19 (based on the referring physicians judgment for patients admitted at the emergency department, taking into consideration onset of symptoms and blood tests); these patients underwent CXR and RT-PCR·testing RT-PCR was used to confirm COVID-19 patients
Wang Hongmei (23)	A BigBiGAN-based architecture was used to train and extract high-dimensional deep learning features of COVID-19 versus non-COVID-19 pneumonia lesions.	Patient data from the First Affiliated Hospital of University of Science and Technology of China and The Lu’an Affiliated Hospital of Anhui Medical University in China were selected (January 18–February 29, 2020). Patients from Stanford University Hospital (February 1–May 30, 2020) served as external validation.	**Inclusion criteria for COVID-19 group**: patients obtained RT-PCR tests to determine COVID-19 status; only those patients who tested positive or negative on at least two RT-PCR tests were included; patients obtained chest CT with or without contrast at time of diagnosis; COVID-19 pneumonia with underlying lung diseases (e.g. lung cancer) were included; **inclusion criteria for non-COVID-19 pneumonia**, as above, patients were included who were clinically suspected to have a viral source of infection; **exclusion criteria:** Tuberculosis, fungal, or bacterial pneumonia patients RT-PCR was used to confirm COVID-19 patients
Chiu (5)	COV19NET was used, which is a DL AI model for the detection of COVID-19 on frontal-view CXR. The algorithm used 2 separate pre-trained networks: (1) an Imagenet pre-trained network based on SE-ResNeXt-50-32x4d tuned on the full ChestX-ray14 data set and (2) the same SEResNeXt-50-32x4d network pre-trained on Imagenet.	The study data sets consisted of one publicly available CXR data set (ChestX-ray14) and two cohort data sets. The training cohort was selected from Queen Mary Hospital (December 31, 2019 and February 15, 2020) and cases across 4 local hospitals in Hong Kong: cases from Queen Mary Hospital, Ruttonjee, Queen Elizabeth and Pamela Youde Nethersole Eastern Hospitals (through March 2, 2020).	**Inclusion criteria**: patients who presented to the Hospital warranting RT-PCR testing and COVID-19 RT-PCR confirmed cased across 4 local hospitals RT-PCR was used to confirm COVID-19 patients
Yang Yanhong (24)	ResUNet was used, which is a DL network using a UNet encoder/decoder back bone, in combination with residual block.	Cases were collected from Guangzhou Eighth People’s Hospital of China (January 6 to April 14, 2020), the Affiliated Hospital of Hebei University of China and the First Affiliated Hospital of Guangzhou Medical University of China.	**Inclusion criteria for COVID-19 group:** patients who tested positive by nucleic acid amplification testing for nasal and pharyngeal swab specimens and were finally confirmed by clinicians and CT imaging; **inclusion criteria for non-COVID-19 group:** patients with tuberculosis, pneumonia and other pulmonary infections Nucleic acid amplification testing was used to confirm COVID-19 patients.

**Legend:** AI: Artificial Intelligence, CT: Computed Tomography, CO-RADS: COVID-19 Reporting and Data System, RT-PCR: Reverse transcription polymerase chain reaction.

**Table 2:** Results.

	Original study authors’ overall conclusions	Sensitivity (%) Confidence Interval (CI) (95%)	Specificity Confidence Interval (95%)	Area Under the Receiver Operator Characteristic curve	Accuracy	Positive Predictive Value	Negative Predictive Value	Others
Lessmann (26)	• Identification of COVID-19 positive patients based on unenhanced chest CT images • Diagnostic performance comparable to radiological observers	Internal Test set: • Eight observer mean sensitivity CO-RADS 5: 61·4% ± 7·9% • AI sensitivity optimal threshold: 85·7% (CI 73·1-98·2%) External Test set: • AI optimal threshold: 82% (CI 69·7-94·3%)	Internal Test Set: • Eight observer mean specificity CO-RADS 5: 99·7% (±0·7%) • AI specificity optimal threshold: 89·8% (CI 79·6-100%) External Test Set: • AI specificity optimal threshold: 80·5% (CI 67·9-93·1%)	• Internal test set AI: 0·95 (CI 0·91-0·98) • External test set AI: 0·88 (CI 0·84-0·93)	n.a.	n.a.	n.a.	n.a.
Zhang Ran (18)	• CV19-Net is able to differentiate COVID-19 disease–related pneumonia from other types of pneumonia, with performance exceeding that of three experienced thoracic radiologists using 500 chest radiographs	• AI using a high-sensitivity operating threshold of 0·4: 88% (CI 87–89%) • Three radiologists with no threshold: 42%, 68%, and 90%	• AI using a high-sensitivity operating threshold of 0·4: 79% (CI 77–80%) • Three radiologists with no threshold: 96%, 85%, and 55%	• For the 500 sampled chest radiographs, CV19-Net: 0·94 (CI 0·93-0·96) • Radiologists: 0·85 (CI 0·81- 0·88) • For the test set, CV19-Net: 0·92 (CI 0·91-0·93)	n.a.	n.a.	n.a.	AI test performance data available according to different age groups and gender
Wang (22)	• The developed AI system might assist radiologists to precisely assess how lesion burden changed over time on CT imaging	• Performance for the identification of changes in lesion burden between two scans: • AI: 0·962 (CI 0·947-1·000) • Radiologist 1: 0·904 (CI 0·872–0·951) • Radiologist 2: 1·000 (CI 1·000–1·000) • Radiologist 3: 0·981 (CI 0·974–1·000)	• Performance for the identification of changes in lesion burden between two scans: • AI: 0·875 (CI 0·833-0·923) • Radiologist 1: 0·979 (CI 0·971-1·000) • Radiologist 2: 0·875 (CI 0·833-0·923) • Radiologist 3: 0·938 (CI 0·917-0·974)	n.a.	Performance for the identification of changes in lesion burden between two scans: • AI: 0·920 (CI 0·900-0·950) • Radiologist 1: 0·940 (CI 0·925-0·962) • Radiologist 2: 0·940 (CI 0·925-0·962) • Radiologist 3: 0·960 (CI 0·950-0·988)	n.a.	n.a.	AI-supported triage improved the efficiency of scan-to-fever-clinician triage at each hospital in the study, ranging from 18·77 min for Tianyou Hospital up to 198·28 min for the Second Xiangya Hospital
Harrison (16)	• AI augmentation significantly improved radiologists’ performance in distinguishing COVID-19 from pneumonia of other origin • Higher measures of accuracy, sensitivity, and specificity for AI when compared with a radiologist-only approach	• AI model: 95% (CI 83–100%, p = 0·001) • Six radiologists’ average: 79% (CI 64–89%, p = 0·001) • Radiologists assisted by the AI model: 88% (CI 74–95%, p < 0·001)	• AI model: 96% (CI 88–99%, p = 0·002) • Six radiologists’ average : 88% (CI 78–94%, p = 0·002) • Radiologists assisted by the AI model: 91% (CI 82–96%, p = 0·001)	AI model: • AUC: 0·95 • AUC of the precision recall curve: 0·90	• AI model: 96% (CI 90–98%, p < 0·001) • Six radiologists: 85% (CI: 77–90%, p < 0·001) • Radiologists assisted by the AI model: average of 90% (CI 83–94 %, p < 0·001)	n.a.	n.a.	n.a.
Mei (28)	• The joint model, the CT model and the clinical model performed equally well in sensitivity compared to the senior thoracic radiologist but showed statistically significant improvement in sensitivity compared to the thoracic radiology fellow	• Senior thoracic radiologist: 74·6% (CI 66·4-81·7%, p = 0·0501) • Thoracic radiology fellow: 56·0% (CI 47·1-64·5%, p = 1 × 10^–4^) • AI joint model: 84·3% (CI 77·1-90·0%)	• Senior thoracic radiologist: 93·8% (CI 88·5- 97·1%, p = 0·005) • Thoracic radiology fellow: 90·3% (CI 84·3-94·6%, p = 0·090) • AI joint model: 82·8% (CI 75·6-88·5%)	• Senior thoracic radiologist: 0·84 (CI 80·0-88·4%) • Thoracic radiology fellow: 0·73 (CI 68·3-78·0%) • AI joint model: 0·92 (CI 88·7-94·8%)	• Senior thoracic radiologist: 80·0% (CI 74·9-84·3%) • Thoracic radiology fellow: 69·0% (CI 64·6-73·0%) • AI joint model: 85·1% (CI 79·3-89·5%)	• Senior thoracic radiologist: 91·7% (CI 85·4-95·5%) • Thoracic radiology fellow: 84·3% (CI 76·1-90%) • AI joint model: 81·9% (CI 75·9-86·7%)	• Senior thoracic radiologist: 84·6% (CI 79·8-88·6%) • Thoracic radiology fellow: 73·8% (CI 68·3-78·9%) • AI joint model: 83·5% (CI 78·6-87·7%)	n.a.
Murphy (20)	• Comparable performance of an AI system in the detection of COVID-19 disease on chest radiographs with that of six independent readers	• At 85% sensitivity, the AI system outperformed all readers for detection of COVID-19	• At 85% sensitivity, AI specificity decreased to 61% (CI 48–72%)	• AI system: area under the ROC curve of 0·81	n.a.	• At a fixed operating point (sensitivity of 75%): • AI system: 77% • Consensus of all six readers PPV: 72%	• At a fixed operating point (sensitivity of 75%): • AI system: 76% • Consensus of all six readers: 78%·	n.a.
Wehbe (17)	• Detection of COVID-19 disease on chest radiographs by the AI algorithm DeepCOVID-XR • Similar performance to that of experienced thoracic radiologists in consensus	• AI: 71% (CI 63–79%, p = 0·78) • Consensus reading: 70% (CI 62–78%, p = 0·78)	• AI: 92% (CI 87–96%, p = 0·29) • Consensus reading: 89% (CI 84–94%, p = 0·29)	• AI: 0·88 (CI 0·84-0·92, p = 0·13) • Consensus reading: 0·85 (CI 0·80-0·89, p = 0·13)	• AI: 82% • Consensus reading: 81%	n.a.	n.a.	• AI algorithm: 300 images analysed in 18 min • Expert radiologist: 300 images analysed in 2·5-3·5 h
Zhou Min (27)	• The trinary scheme achieved specialist-level performance on patient-level classification	Test set: • Trinary scheme: 88·9% External validation set: • Trinary scheme: 86% External validation set: • 5 specialists mean: 71·2% (57·8 −84·7%) • 5 residents mean: 57·2% (51·3-63·1%)	Test set: • Trinary scheme: 94·4% External validation set: • Trinary scheme: 77·2% External validation set: • 5 specialists mean: 89·2% (80·5-97·9%) • 5 residents mean: 76·8% (68·8-84·8%)	Test set: • Trinary scheme: 0·95 External validation set: • Trinary scheme: 0·87 Radiologists: • Specialist group: 0·802 • Resident group: 0·67	Test set: • Trinary scheme: 91·7%	n.a.	n.a.	n.a.
Castiglioni (21)	• This preliminary experience based on ten CNNs trained on a limited training dataset shows an interesting potential of deep learning for COVID-19 diagnosis	• Deep learning model (independent dataset): 0·80 (CI 0·72-0·86, p < 0·005) • Radiologist 1 (independent dataset, Centre 1): 0·64 (CI 0·52-0·74) • Radiologist 2 (independent dataset, Centre 2): 0·64 (CI 0·52-0·74)	• Deep learning model (independent dataset): 0·81 (CI 0·73-0·87, p < 0·005) • Radiologist 1 (independent dataset, Centre 1): 0·78 (CI 0·61-0·90) • Radiologist 2 (independent dataset, Centre 2): 0·86 (CI 0·71-0·95)	• Deep learning model (independent dataset): 0·81 (CI 0·73-0·87)	n.a.	n.a.	n.a.	n.a.
Wang Hongmei (23)	• Human expert diagnostic performance improved using a combined deep learning-radiomics model	• 3 radiologists’ average on two test datasets: 74·7%· Radiologists’ average using deep learning and radiomics features: 91·2%	• 3 radiologists’ average on two test datasets: 80·3% Radiologists’ average using deep learning and radiomics features: 91·9%	Training dataset: Linear classifier: 0·98 (91·8% sensitivity, 93·4% specificity) vs. 0·91 (sensitivity 80·0%, specificity 87·2%) External validation dataset: Linear classifier: 0·84 (sensitivity 75·7%, specificity 76·8%) vs. 0·86 (sensitivity 76·5%, specificity 80·9%)	n.a.	n.a.	n.a.	n.a.
Chiu (5)	• AI model outperformed radiologists in a prospectively collected COVID-19 screening validation cohort for the detection of COVID-19, and also in distinguishing COVID-19 on CXR from non-COVID-19 respiratory infectious agents	DL algorithm: 0·85 (p = 0·01) • RT-PCR as reference standard Radiologists: 0·49 (p = 0·01) • RT-PCR as reference standard	DL algorithm: 0·72 (p = 0·63) • RT-PCR as reference standard Radiologists: 0·74 (p = 0·63) • RT-PCR as reference standard	DL algorithm: • AUC: 0·81 (CI 0·75-0·87, p < 0·001) • RT-PCR as reference standard Radiologists: • AUC: 0·62 (CI 0·54-0·69, p < 0·001) • RT-PCR as reference standard	n.a.	DL algorithm: 0·55 (p = 0·14) • RT-PCR as reference standard Radiologists: 0·44 (p = 0·14) • RT-PCR as reference standard	DL algorithm: 0·92 (p = 0·01) • RT-PCR as reference standard Radiologists: 0·78 (p = 0·01) • RT-PCR as reference standard	n.a.
Yang Yanhong (24)	• A deep learning algorithm-based AI model improved radiologists’ performance in distinguishing COVID-19 from other pulmonary infections using chest CT images	• Radiologists’ average: 0·895 (CI 0·845-0·934) • Radiologists’ average with AI assistance: 0·942 (CI 0·896-0·968) • Only AI: 0·918	Only AI: 0·909	Final model: • AI: 0·903 Test set: • Human readers only: 0·963 • AI-supported reading: 0·966	• Radiologist: 0·941 (CI 0·896-0·968) • Radiologist with AI assistance: 0·951 (CI 0·909-0·976) • Only AI: 0·914	Only AI: 0·898	Only AI: 0·928	n.a.

Legend: Legend: AI: Artificial Intelligence, CT: Computed Tomography, CXR: Chest X-Ray, CO-RADS: COVID-19 Reporting and Data System, RT-PCR: Reverse transcription polymerase chain reaction, AUC: area under the curve, ROC: receiver operating characteristic, CI: Confidence Interval, n.a.: not applicable.

## 3 Results

**Fig. 1:**
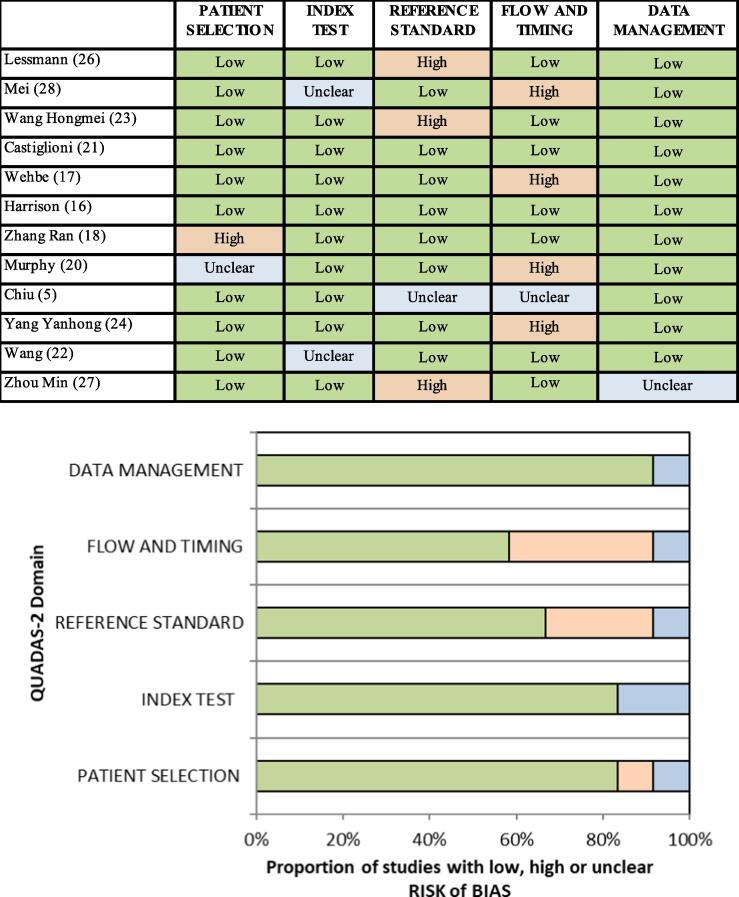

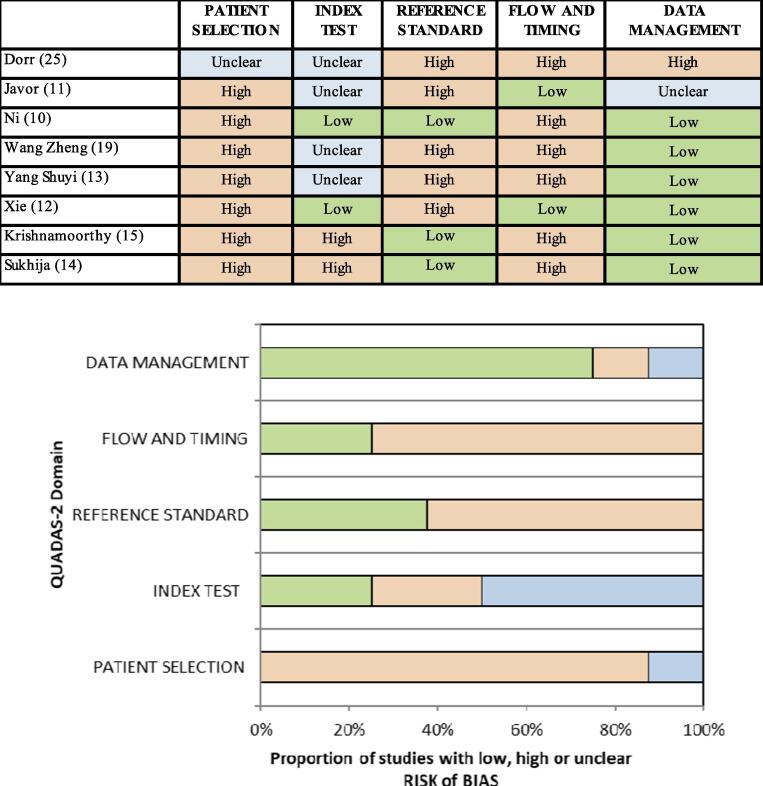
(a) QUADAS-2 assessment results for included studies. Legend: QUADAS 2 assessment results for included studies, indicating low (green colour), high (orange colour) or unclear (blue colour) risk of bias for the relevant domains. (b) QUADAS-2 assessment results for excluded studies. Legend: QUADAS 2 assessment results for excluded studies, indicating low (green colour), high (orange colour) or unclear (blue colour) risk of bias for the relevant domains. (For interpretation of the references to colour in this figure legend, the reader is referred to the web version of this article.)

**Fig. 2:**
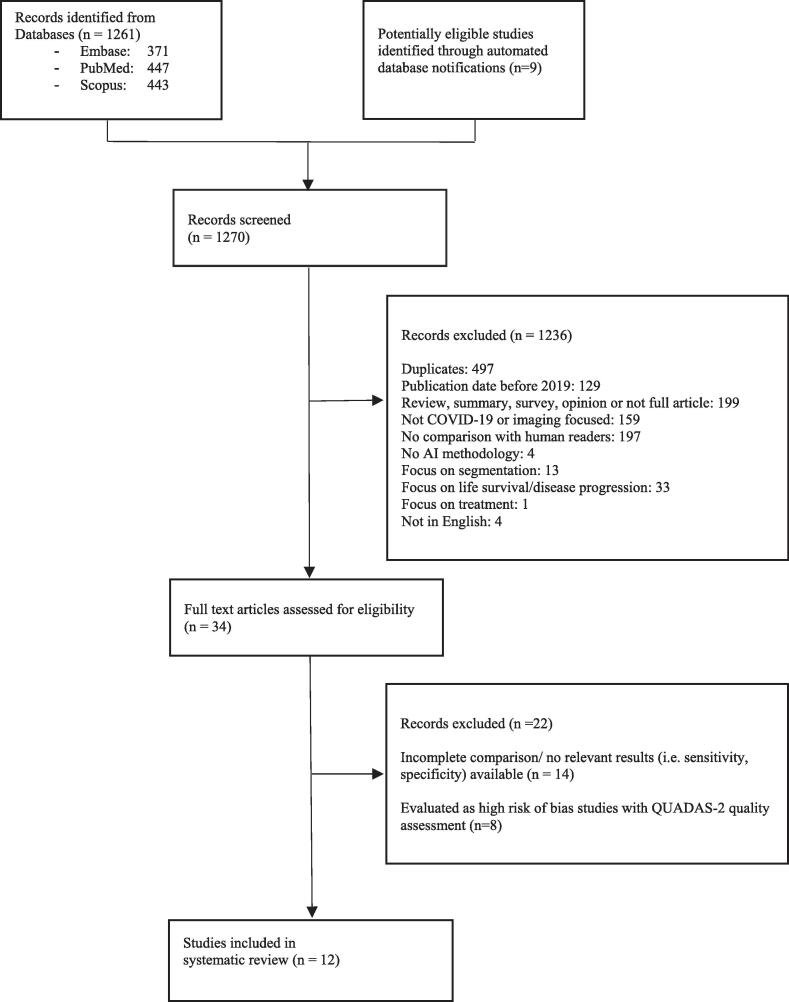
Study selection.

The search strategy identified a total of 1261 articles on November 30, 2020, with a further six studies identified via database notifications of the same search until January 31, 2021. Overall, 771 articles were screened for meeting the selection criteria. A total of 20 studies that met these criteria were assessed for the risk of bias with the QUADAS-2 tool [5], [10], [11], [12], [13], [14], [15], [16], [17], [18], [19], [20], [21], [22], [23], [24], [25], [26], [27], [28]. Eight studies with a high risk of bias rating in at least two domains were excluded (Fig. 1, appendix, Table 3). A total of 12 studies were included in our systematic review [5], [16], [17], [18], [20], [21], [22], [23], [24], [26], [27], [28]. (See Fig. 2 for the study selection and appendix, table 4 for the PRISMA-DTA checklist and appendix, table 5 for information on statistical analysis).

**Fig. 3:**
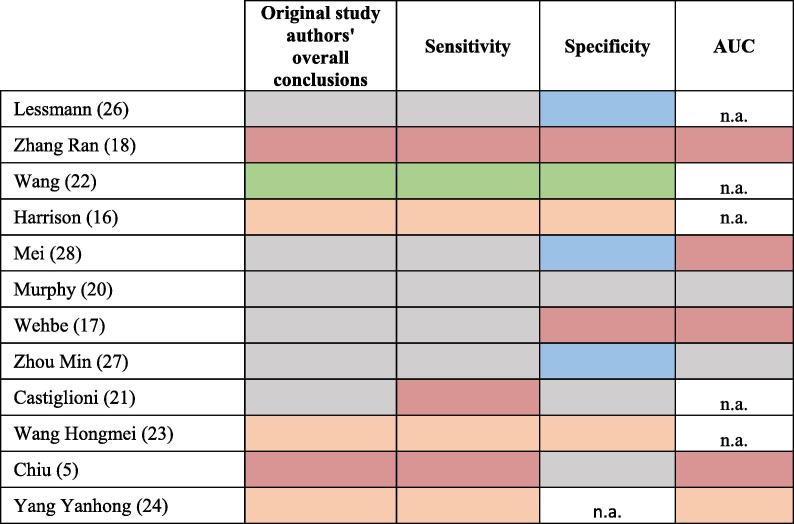
Overall results visualization. Legend: Red colour indicates “Better performance of AI”, blue colour indicates “Better performance of human readers”, orange colour indicates “Increased performance of human readers with AI support”, grey colour indicates “Comparable performance of AI and human readers to differentiate COVID-19”, green colour indicates “Comparable performance of AI and human readers for lesion changes”, no colour and “n.a.” indicates “Values in comparison not indicated” (For interpretation of the references to colour in this figure legend, the reader is referred to the web version of this article.)

### 3.1 Study characteristics

We identified that seven studies used computed tomography (CT) as the main imaging modality [16], [22], [23], [24], [26], [27], [28], with the remaining five studies focusing on chest X-ray (CXR) imaging [5], [17], [18], [20], [21]. (See Table 1a for details on study characteristics). All studies applied a DL model for their AI methodology that included neural networks (Table 1b). For the majority of four studies, China was indicated as the country where the imaging took place [23], [24], [27], [28], with two studies using imaging data from both China and the United States [16], [23]. In terms of data source origins, imaging data was considered from various geographical locations, with a focus on patients from the United States for two studies [17], [18], two studies from the Netherlands [20], [26], one study from Italy [21], and one study from Hong Kong [5].

There was considerable heterogeneity between the study designs, data collection and patient selection criteria applied throughout the studies, i.e. with different inclusion criteria for the selection of COVID-19 positive patients and the related characteristics for imaging required, as well as the use of automatic assessment scoring and subgroup analysis.

For the reference standard indicated in the included papers, three studies used RT-PCR [20], [21], [28], three studies applied consensus findings between readers [13], [16], [18], two studies provided outcome values for both these options [17], [19], and in four studies the use of the reference standard was not sufficiently detailed, but included the above (see Table 1a) [5], [23], [26], [27]. All studies were based on patient selection with the diagnosis of an RT-PCR or nucleic acid amplification (NAAT) test. The total number of human readers ranged from two readers to a panel of ten, with practice levels ranging from less than five years [26], to over 30 years of experience in thoracic imaging [20].

In ten studies, various hospitals, academic centres and clinics were indicated as the data source [16], [17], [18], [21], [22], [23], [24], [26], [27], [28]. Two studies used a mix of data from publicly available databases as well as from hospitals [5], [20]. There was a large variety of patients in the total numbers used in the overall datasets, ranging from 216 to 25,146 patients. Studies that used CXR as the imaging modality had generally larger sample sizes. All studies used differentiated datasets for training, testing and validation (Table 3). Ten studies provided performance measurements for testing datasets, reporting 15 different sample sizes between 18 and 2,193 patients [16], [17], [18], [20], [21], [22], [23], [24], [26], [27]. Four studies reported values for validation, with sample sizes ranging between 18 and 910 patients [5], [22], [23], [27]. While three studies provided performance measurements for external validation [22], [23], [27], one study did this for independent internal validation [5].

**Table 3:** Overview of datasets used for statistical analysis.

**Legend:** The sample sizes for patients for the different datasets in this figure may show discrepancies to the ones reported in table 1a) Study characteristics. This is due to original studies subdividing datasets for different purposes in the datasets in the context of sampling and splitting data. In addition, sample sizes may vary for the statistical analysis, where performance measurements are not always consistently provided for all datasets in the original studies. Numbers highlighted in red did not provide related performance measurements.

In eight out of 12 studies, there was a larger representation of men in the patient characteristics [16], [20], [21], [23], [24], [26], [27], [28], ranging from 54 to 59% of males [28], [23], of the total number of patients, and up to 65.8% for specific datasets used for training [24]. Three studies reported equal numbers of males and females [5], [18], [22]; and with 53%, one study indicated a larger representation of women in the patient characteristics [17].

Age values were reported in a heterogeneous way across the studies. Three studies reported mean ages for the total patient population [17], [20], [28], ranging from 40.7 [28] to 58 years [17]. Three studies reported mean ages for COVID and non-COVID groups [14], [16], [19], with one study indicating a younger average age of 48 years in the COVID group vs. 62 years in the non-COVID group [16], and two studies reporting more balanced ages for the two groups [18], [21].

### 3.2 Performance outcomes

**Fig. 4:**
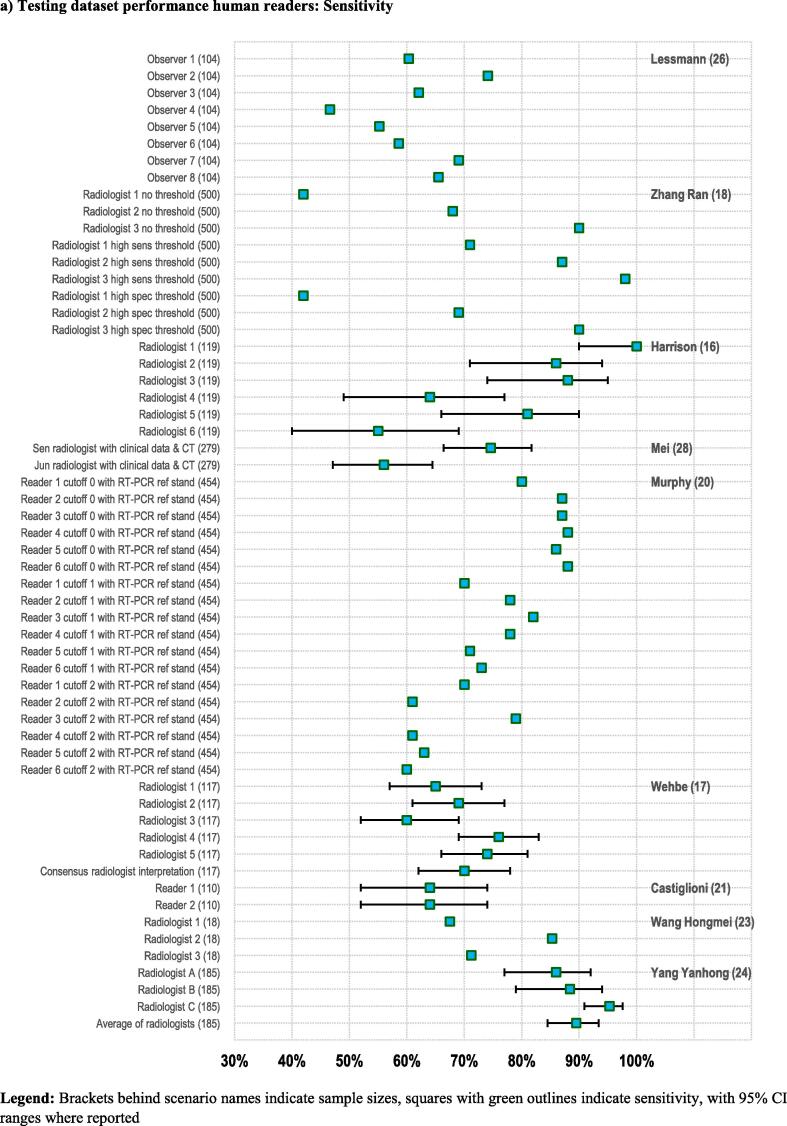

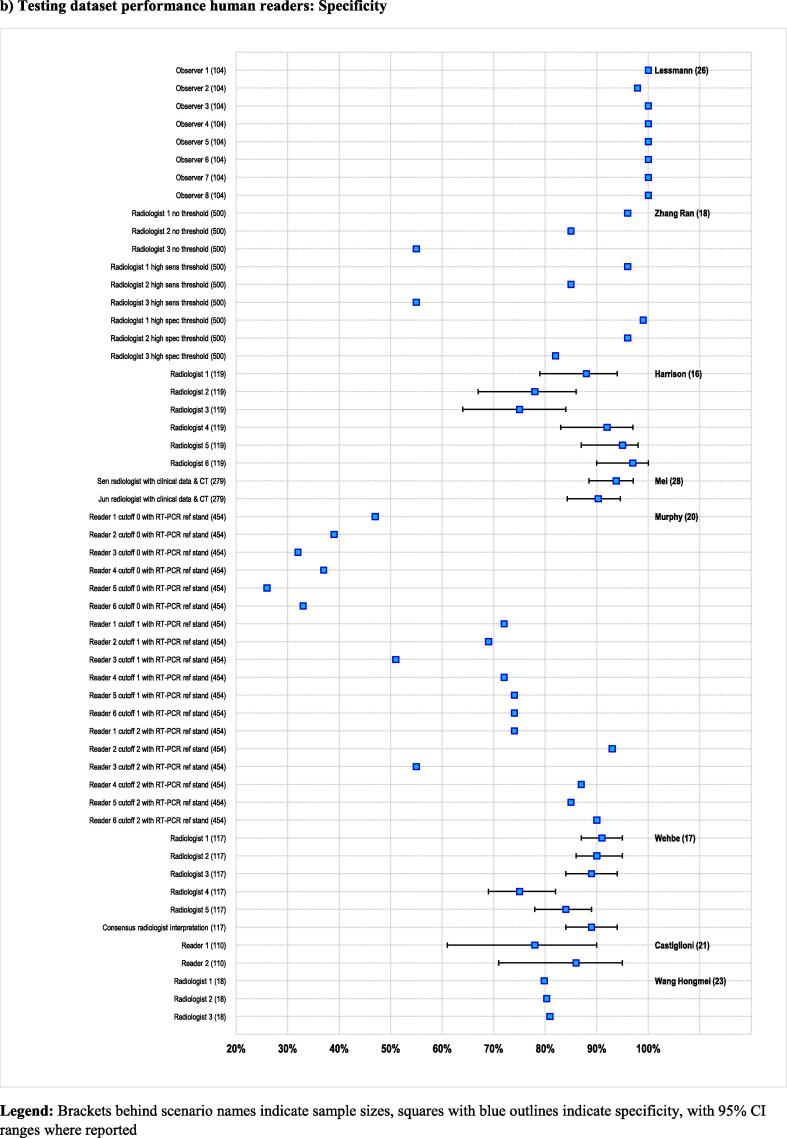

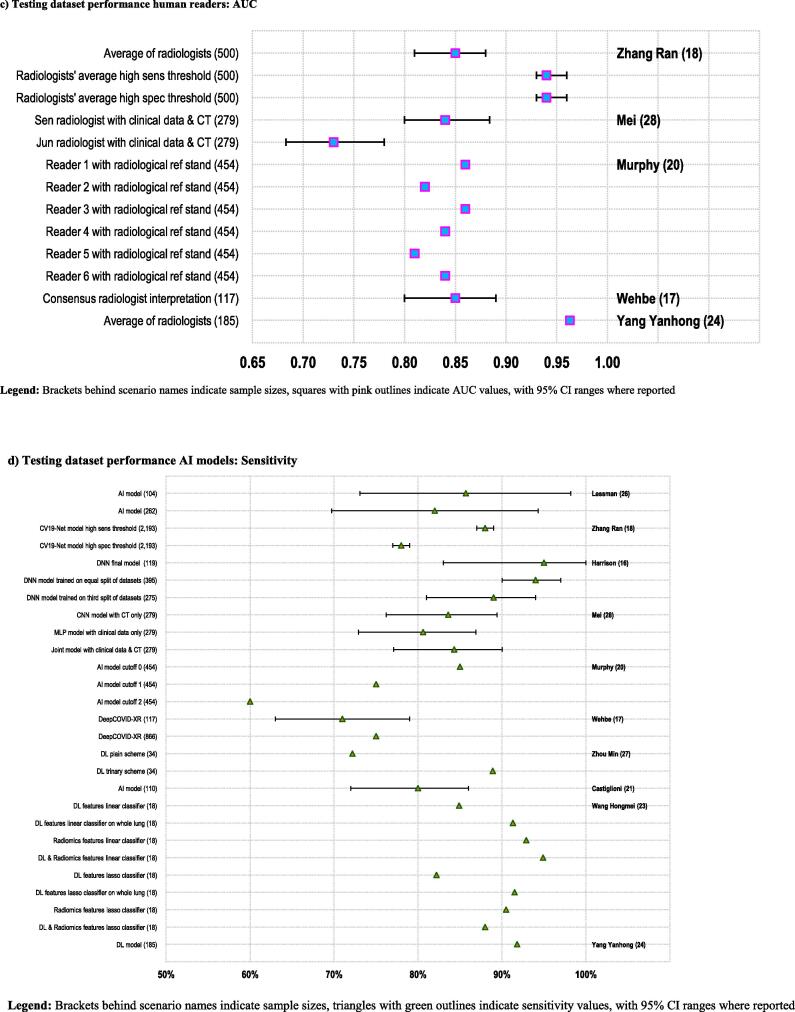

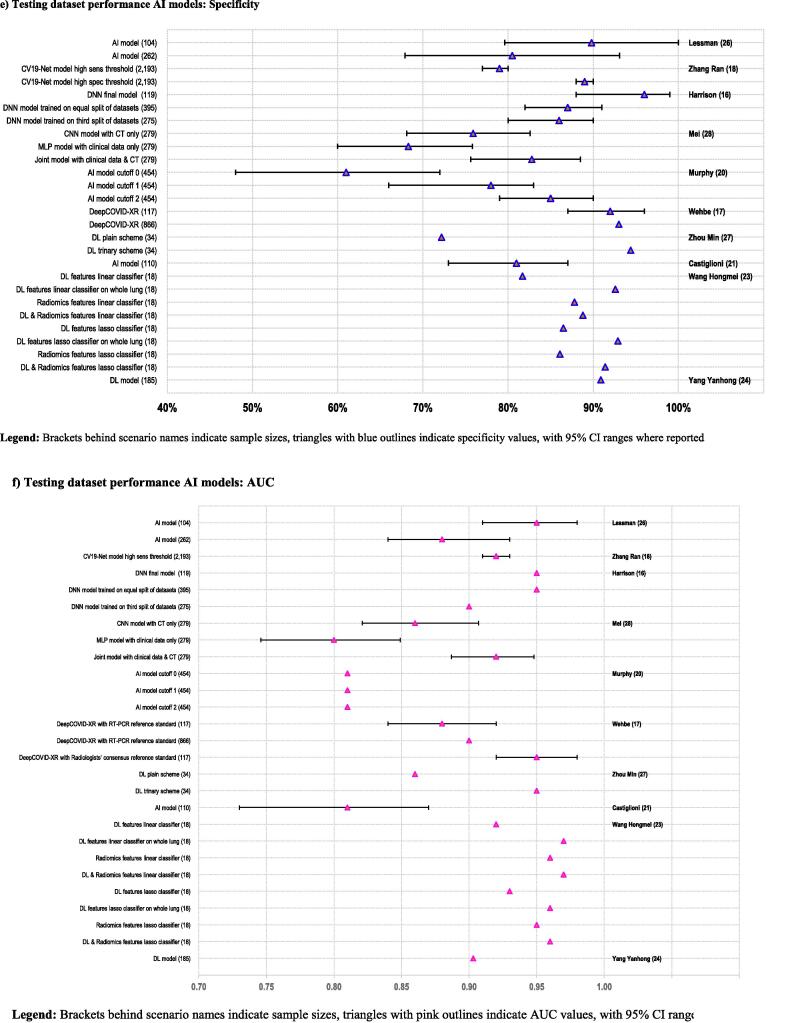

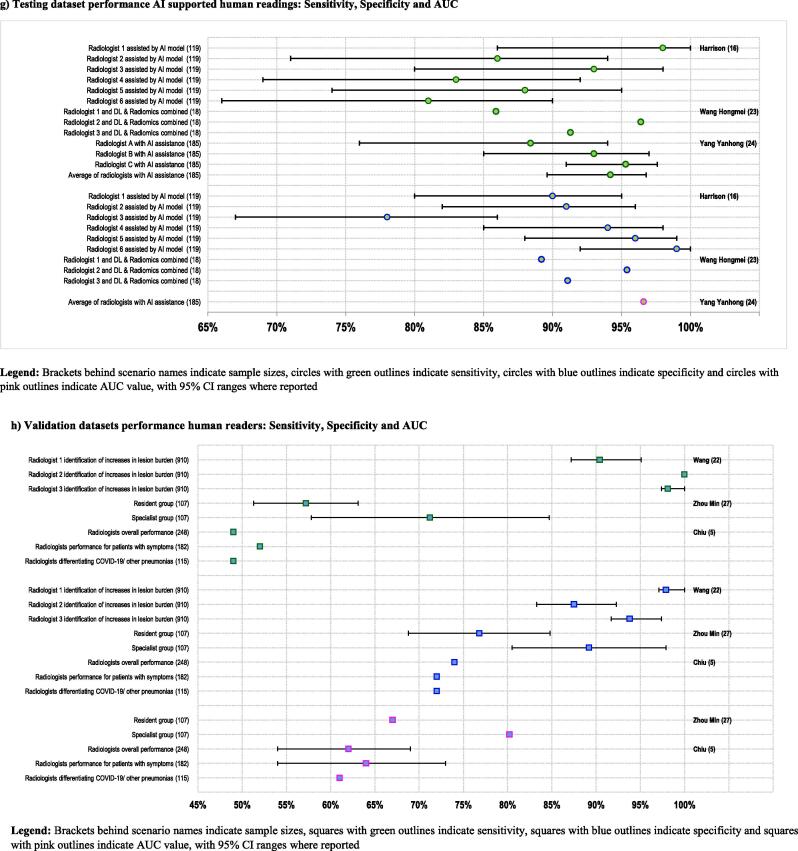

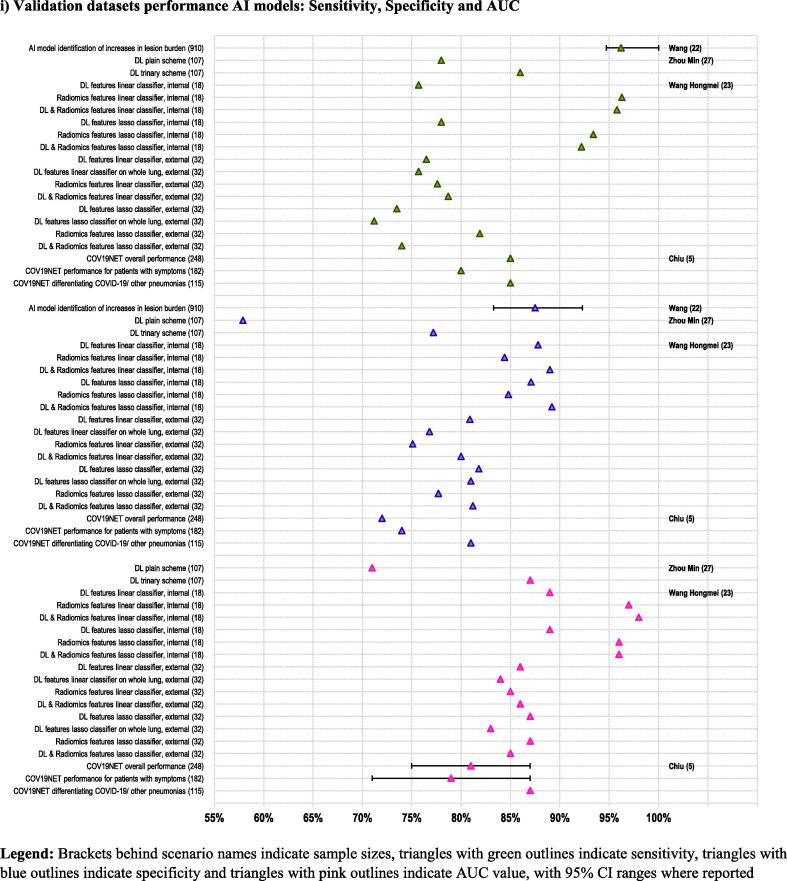
Forest plots with performance overviews.

**Fig. 5:**
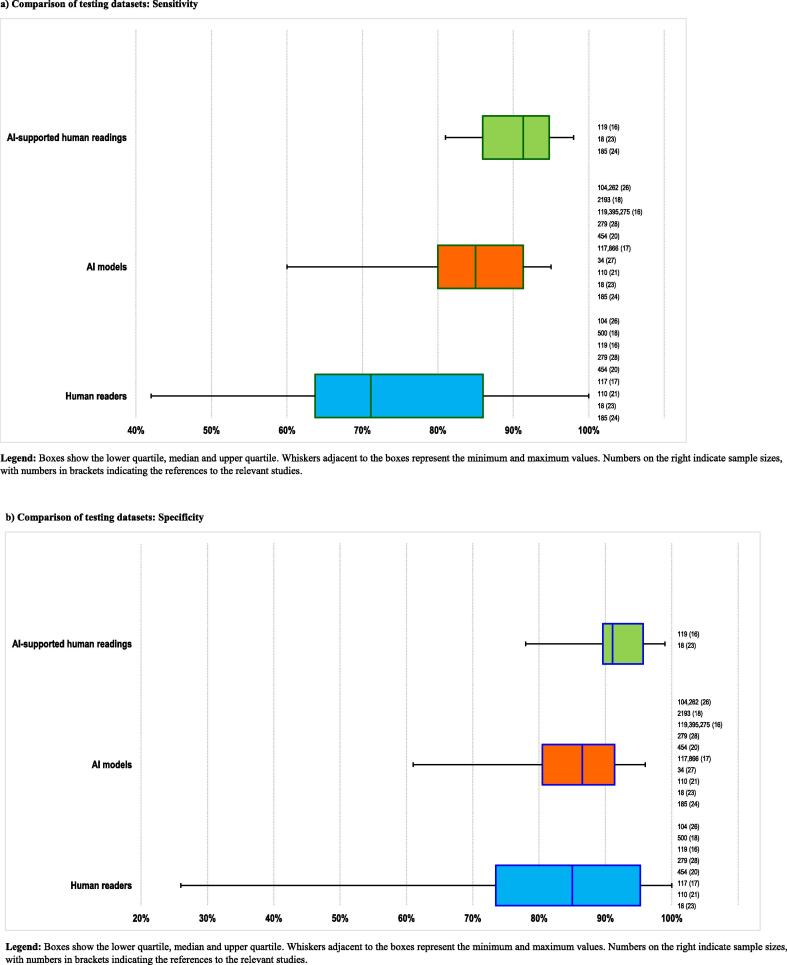

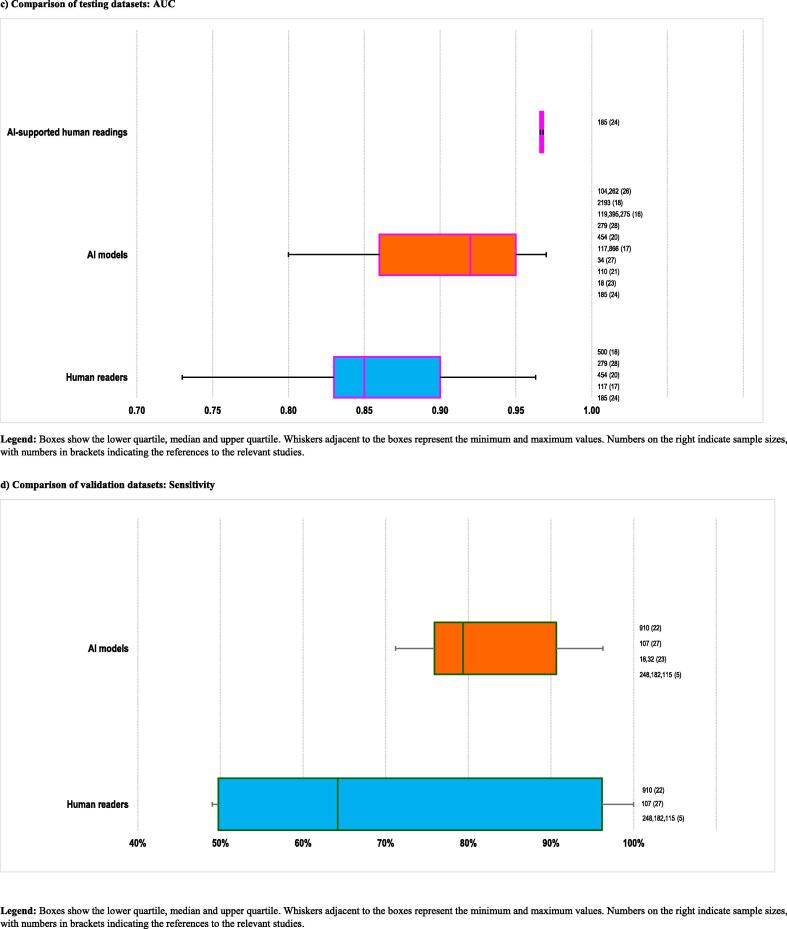

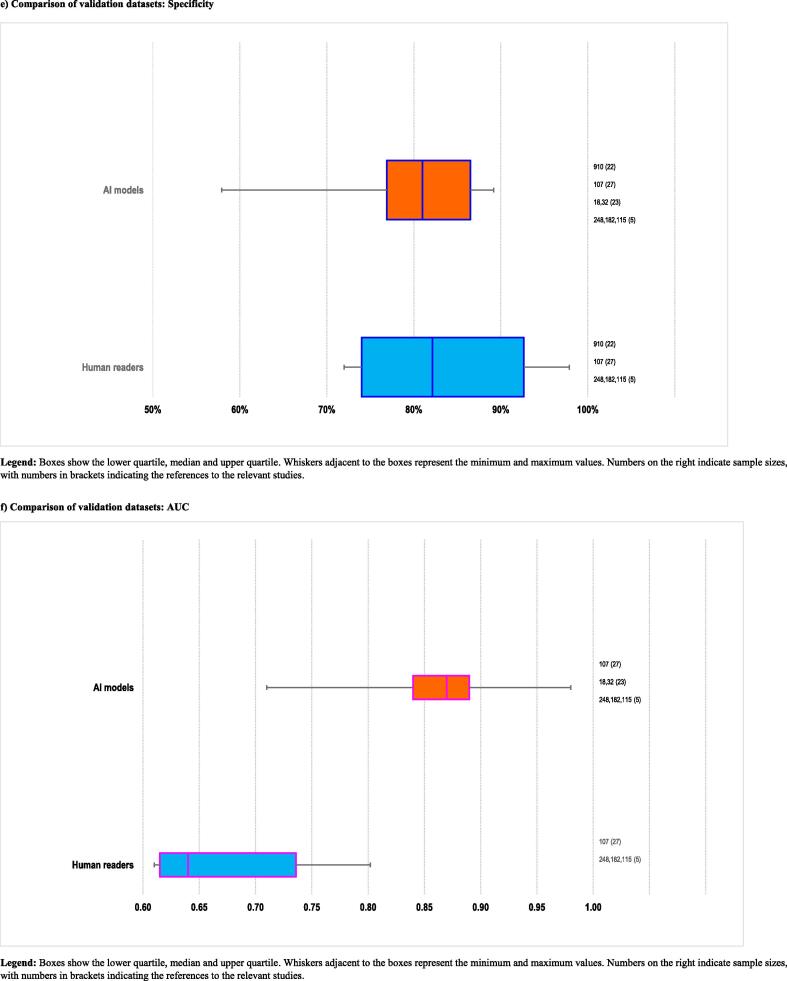

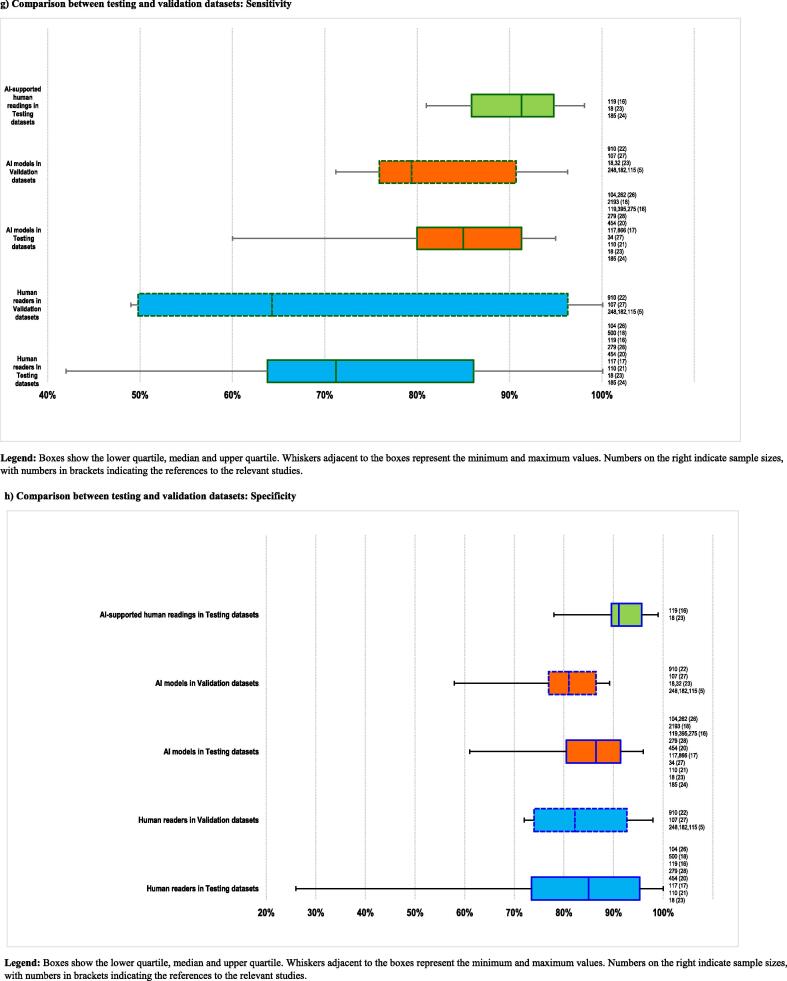

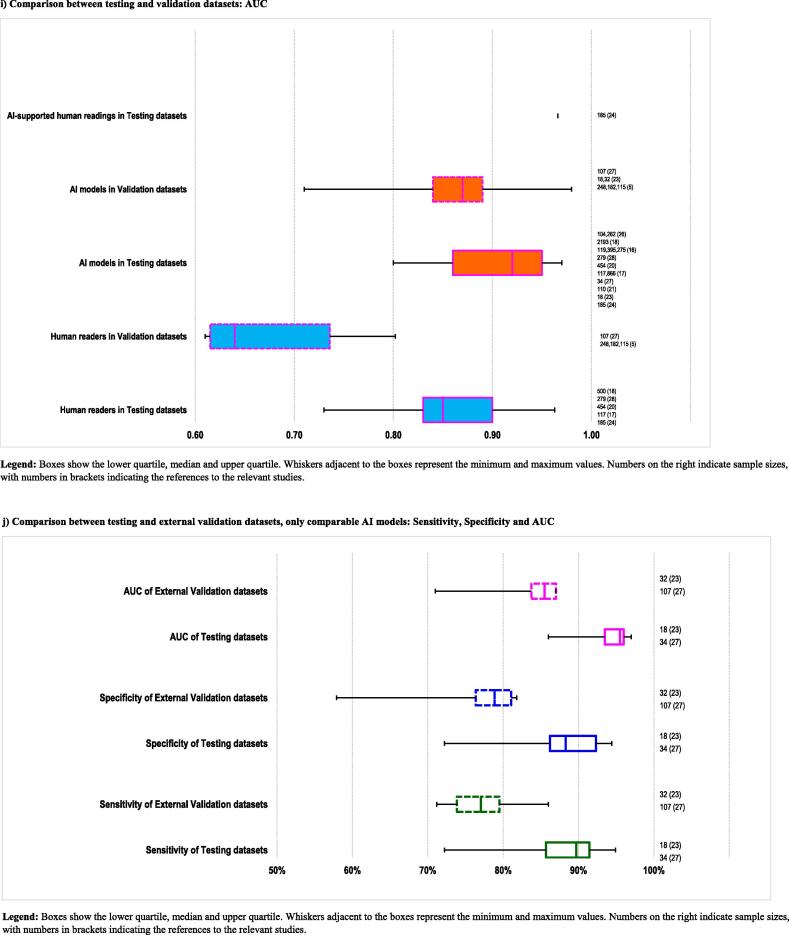
Box plots with performance overview comparisons.

We focused on the performance data from 126 scenarios, reporting sensitivity (118 for specificity) in 12 studies, and 65 scenarios for AUC values in 11 studies, applying our grouping of the categories into human readers, AI models and AI-supported human readings, visualised in Fig. 4. (See Fig. 5 for a comparison across different datasets and Table 4 for an overview of the quantitative results).

**Table 4:** Overview of statistical analysis results (Min/Max, Median, IQR).

**Legend:** IQR: Interquartile range, *one value only, n.a.: not applicable.

### 3.3 General findings

For overall conclusions, as claimed by the authors in the 12 original studies, six reported a diagnostic performance of the AI model which was comparable to human readers, with three focused on CT [26], [27], [28], and three with CXR as an imaging modality (Fig. 3 and Table 2) [17], [20], [21]. Two other studies using CXR indicated that their AI model outperformed human readers [5], [18]. Compared to a reader-only approach, three studies reported that AI augmentation improved human readers’ performance using CT as an imaging modality [16], [23], [24]. One study reported positive results of the AI system to aid radiologists in the assessment of changes of the lung lesion burden on pairs of CT scans, with comparable performance of the AI model to human readers [22].

### 3.4 Sensitivity

#### 3.4.1 Testing datasets

Ten of the studies reported sensitivity with 42–100% for human readers, and 60–95% of AI models [16], [17], [18], [20], [21], [23], [24], [26], [27], [28]. Three studies examined AI-supported human readings, with sensitivity values from 81 to 98% [16], [23], [24]. Median sensitivity was the highest for AI-supported human readings (91.3%), with lower values observed for AI models (85%) and human readers (71.1%). The interquartile range (IQR) of the above studies was lowest for AI-supported human readings (8.8%) and AI models (11.3%), with a higher value for human readers (22.3%).

#### 3.4.2 Validation datasets

Three of the studies reported sensitivity at 49–100% for human readers [5], [22], [27], with four studies indicating ranges of 71.2–96.3% for AI models [5], [22], [27]. No values were provided for AI-supported human readings. Median sensitivity was higher in AI models (79.4%) than for human readers (64.2%).The IQR of the above studies was lower in AI models (14.8%) than for human readers (46.4%).

### 3.5 Specificity

#### 3.5.1 Testing datasets

Eight studies reported specificity with 26–100% for human readers [16], [17], [18], [20], [21], [23], [26], [28], while ten reported values of 61–96% for AI models [16], [17], [18], [20], [21], [23], [24], [26], [28]. Two studies examined AI-supported human readings, with specificity values from 78 to 99% [16], [23]. Median specificity was highest for AI-supported human readings (91.1%), with lower values observed for AI models (86.5%) and human readers (85%). The IQR of the above studies was lowest for AI-supported human readings (6.1%) and AI models (10.9%), with a higher value for 21.8% for human readers.

#### 3.5.2 Validation datasets

Three of the studies reported specificity with 72–97.9% for human readers, and 57.9–89.2% for AI models [5], [22], [27]. No values were provided for AI-supported human readings. Median specificity was 81% for AI models and 82.2% for human readers. The IQR of the above studies was lower in AI models (9.6%) than for human readers (18.7%).

### 3.6 AUC values

#### 3.6.1 Testing datasets

Five of the studies reported AUC ranges of 0.73–0.963 for human readers [17], [18], [20], [24], [28], with ten studies indicating ranges of 0.80–0.97 for AI models [16], [17], [18], [20], [21], [22], [23], [26], [28]. One study examined AI-supported human readings, reporting an AUC of 0.966 [24]. AI models had median values of 0.92 and human readers of 0.85. The IQR of the above studies was 0.07 for human readers and 0.09 for AI models.

#### 3.6.2 Validation datasets

Two studies reported AUC ranges of 0.61–0.802 for human readers [5], [27], with three studies indicating values of 0.71–0.98 for AI models [5], [23], [27]. No values were provided for AI-supported human readings. Median AUC values were higher in AI models (0.87) compared to human readers (0.64). The IQR of the above studies was lower for AI models (0.05) than for human readers (0.121).

### 3.7 Comparison of performance between testing and validation datasets

Only scenarios for AI models and human readers were comparable between testing and validation datasets. AI-supported readings showed the highest median values, and in general, the median for sensitivity, specificity and AUC values, was higher in the testing datasets. The IQR for sensitivity in general was lower in the testing datasets, while being comparable for specificity. Only for comparable datasets in two studies, related to testing and external validation, the medians in the testing datasets were higher, showing an overall reduced performance in external validation (Fig. 5 j).

### 3.8 Time savings

AI-supported triage improved the efficiency of scan-to-fever-clinician triage at each hospital in the study by Wang and colleagues, with a median reduction in triage time ranging between 18.77 and 198.28 min in different hospitals [22]. Wehbe and colleagues reported that the time to analyse a data subset with AI took approximately 18 min, compared to approximately 2.5–3.5 h for each radiologist [17].

## 4 Discussion

Our study shows promising performance results of AI-supported detection of COVID-19 imaging, specifically comparing the performance of AI and human readers, however these need to be interpreted in the context of risks of bias. The medians of all performance values in the testing datasets were in general the highest for AI-supported human readings, followed by AI models, and then human readers alone, as shown in Table 4. However, human readers alone reached the highest maximum, but also the lowest minimum values in testing datasets, with the latter especially notable for specificity. Variability of diagnostic performance focused on the IQR was in general lower in AI systems or AI-supported readings. In addition, some studies reported time savings with AI models, as detailed in Table 2. Reporting related to speedier analysis with AI also included AI-supported triage, whereby time-to-triage was faster compared to a standard clinical workflow across different clinics. Notably, this implied an ideal scenario where clinicians would respond instantly to AI notifications and could thereby potentially shorten the time to diagnosis, with multiple benefits for the isolation and the treatment of affected patients [22].

In the validation datasets, there was an overall reduction in performance in comparison to the testing datasets related to median values, as shown in Fig. 5. However, a detailed analysis of the same performance measurements across all studies was difficult, as most studies did not include a complete reporting on all performance measurements disaggregated by datasets and differentiating all scenarios for human readers, AI models and AI-supported readings. The fragmented reporting related to performance measurements is detailed in the supplementary appendix, table 5. Notably, there was large heterogeneity in the methodology and selection criteria applied, including patient characteristics and sample sizes, as well as levels of patient detail disaggregation (see Table 1a and 1b). Most of the studies in our review focused on differentiating COVID-19 pneumonia from other types of pneumonia, showing a potential added value of imaging as a supplementary diagnostic measure, in the context of fast and accurate reporting and earlier identification of potentially infected COVID-19 patients. However, there were differences in how studies attempted to evaluate these merits, i.e. through a comparison of AI and human readers as well as AI-supported readings [16], [23], [24], versus human readings alone [5], [17], [18], [20], [21], [22], [26], [27], [28]. The studies also applied different strategies regarding subgroup analysis, and the differentiation of training, (external) testing and (external) validation. In order to ensure the robustness of final results, future studies should clearly describe the splitting of all datasets for training, testing and validation, not only subsets of these (i.e. training and validation only). In addition, the documentation of relevant patient characteristics should be consistently applied for all datasets. A variety of different approaches were used for the scenarios of human readers, including different levels of experience, as well as thresholds and cut-off points (see Fig. 4). The heterogeneity in scenarios also applies to AI models, which reported different model types, design features, classifiers and reference standards. Due to such heterogeneity in the study methodology and analysis applied in the different studies and the resulting lack of comparability, it is difficult to make solid conclusions regarding improved performance measurements with AI. There were also major differences in baseline characteristics between patient groups with COVID-19, and those with non–COVID-19 pneumonia, introducing possible selection bias with imbalances regarding gender and age, with a related overrepresentation of younger males, including also different datasets within a study as shown in Table 1a. In addition, reporting on the details of patient characteristics for different cohorts varied, for example there was limited information on patients who may have been immunosuppressed.

Notably, some CT-focused studies featured smaller sample sizes for certain datasets – mostly for validation [23], [27], and patients with early-stage COVID-19. The limitations related to patient populations were further complicated by variations in image quality, and heterogeneity in imaging acquisition and post-processing parameters as shown in our QUADAS-2 assessment. Future studies should focus on testing AI models with data from more populations and geographical areas. Imaging should also include more data and information on disaggregation related to all ethnicities, and not limited to Caucasian and Asian races [21], as well as older patients, with more equal gender representation to allow for further generalisations. To assess and validate the robustness of AI models, training with larger multi-centre datasets and consistent external validation is required, as well as more prospective study data and evaluations [26], [29].

Overly positive interpretations of promising performance results attributed to AI models or AI-supported readings should be treated with caution, as there is a risk of overestimating results due to several potential confounding factors. As such, Chiu and colleagues have discussed how the use of RT-PCR as the ground truth for training models may not reflect the real performance of AI systems, since false-negative rates of RT-PCR have been reported to be as high as 30% [5]. In addition, there is a potential susceptibility of AI systems to ‘learn’ dataset characteristics instead of disease classification, for example by introducing a bias described as ‘shortcuts learning’ [30]. In these instances, models may rely on features that are not related to correct object classification for a disease pathology, by relying on differences in the background instead, such as textual markers in obtained images, for example related to patient positioning [31]. Another area of attention is related to an intrinsic feature of AI, specifically its “black box” character: it is difficult to rule out that an algorithm is not using findings outside the lungs when discriminating for COVID-19 [5]. The black box problem emphasizes another value of human readers, namely that results are difficult to explain [27]. Moreover, human analysis is required to rule out motion artefacts which may cause errors when detecting COVID-19 [26].

Our QUADAS-2 analysis highlighted the need to address possible risk of bias in future studies, especially related to a clear need for adequate and consistent descriptions of patient selection and study population description. Further areas of attention are clear information on the use of index tests, reference standards and flow and timing between these. In the context of imaging, detailed descriptions of imaging acquisition and processing methods are required. In addition, clear descriptions of data sources are important. An analysis of the statistical methods applied in the studies revealed further methodological concerns, such as the inconsistent use of 95% confidence intervals for all performance values, or of p-values for interpreting lack of significance, as shown in Table 5 of the supplementary appendix. We found considerable limitations concerning the methods applied to compare performance between AI systems and human readers. For example, the selection of sample sizes can strongly influence performance measurements and only one study analysed the threshold above which no performance gain could be obtained [18]. Several studies did not consistently apply the agreement index, using instead, average values of reader performance. To enable appropriate inter-rater agreement, studies should focus on *agreement* rather than *correlation* indices [32], [33]. In addition, future statistical analysis should consistently analyse the disease prevalence, for example, by using Negative Predictive Value and Positive Predictive Value parameters, as well as positive and negative likelihood ratios to reduce uncertainties regarding the validity of diagnostic tests [34], with the latter ratios only reported in one of the 12 studies [21]. In this way, meaningful analysis of the overall merit of AI-supported imaging-based COVID-19 detection beyond high-prevalence populations could be ensured.

Future studies need to address concerns of AI methodology regarding transparency, reproducibility, ethics and effectiveness, as well as specific reporting standards for AI studies [35]. This includes attention to critical elements that are required for coherent use of methodology, including study design, clear description of data sets and patient characteristics as well as consistent application of assessment methods that analyse potential risks of bias.

We acknowledge certain limitations in our study. We only included peer-reviewed studies in English from the published literature, potentially increasing the likelihood of publication bias influencing our findings. We did not evaluate the methodology of the AI and DL approaches applied in the reported studies, which may further affect the risk of bias. Importantly, we were unable to provide a *meta*-analysis of the studies included in our review, due to a large heterogeneity in the methodologies and results of the studies, precluding conclusions on pooled diagnostic accuracy of AI versus human readers.

## 5 Conclusions

While the studies included in our systematic review reported promising results of AI, these need to be seen in the context of COVID-19 severity, overall disease prevalence in a population, as well as several risks of bias regarding study methodologies applied. For early-onset patients and those who are asymptomatic or have mild disease, the performance of imaging may not be satisfactory. AI-supported imaging would therefore be most promising in high prevalence areas, and as a support tool for rapid diagnosis to identify suspected patients as a priority within a triage setting [17], [21], [28]. At the same time, the involvement of human readers remains crucial.

Our study identified and highlighted several inconsistencies of data reporting and presentation, heterogeneity for study methodologies and evaluation methods applied, certain areas of risk of bias (e.g. selection bias, “shortcut learnings” bias) and limitations of statistical analyses.

While our study presented an overall promising potential of AI models for COVID-19 imaging diagnostic decision-making, this can only be confirmed by future studies with an improved and harmonised overall methodology that allows for reliable generalisability of results. In this context, we support efforts for the development and future implementation of a set of methodological ‘critical elements’ regarding study design, and assessment methodology to facilitate data aggregation, comparability and conclusions of the possible added value of AI-based imaging systems for diagnostic decision-making.

## Declaration of Competing Interest

The authors declared that there is no conflict of interest.
