# Supplementary material for: Artificial intelligence for imaging-based COVID-19 detection: Systematic review comparing added value of AI versus human readers
Source: Eur J Radiol. 2021 Dec;145:110028. doi: 10.1016/j.ejrad.2021.110028 (PMC8594127; doi:10.1016/j.ejrad.2021.110028)
Supplement: Supplementary data 1 [file mmc1.doc]

**Supplementary Appendix**

**Table 1: Selection Criteria**

**Inclusion criteria:**

- AI imaging study focused on COVID-19 detection or diagnosis
- Artificial intelligence methods include machine learning (ML) and deep learning (sub-branch of ML)
- Element of comparison with human readers

**Exclusion criteria:**

- Not focused on COVID-19 lung imaging (ex-vivo imaging and pathology studies)
- No sufficient comparison element with human readers, including relevant outcome result reporting (i.e. sensitivity and specificity)
- Systematic review, overview, summary, survey or opinion paper, not full paper
- Focus on segmentation and features extraction, for example locating objects or boundaries
- Focus on COVID-19 Treatment
- Life expectancy/survival and disease risk prediction
- Duplicate
- Not in English

The study protocol has not been registered.

**Table 2 Search strings**

**PubMed search string:**

(("artificial intelligence"[MeSH Terms] OR ("artificial"[All Fields] AND "intelligence"[All Fields])) OR "artificial intelligence"[All Fields]) OR (("machine learning"[MeSH Terms] OR ("machine"[All Fields] AND "learning"[All Fields])) OR "machine learning"[All Fields]) OR (("deep learning"[MeSH Terms] OR ("deep"[All Fields] AND "learning"[All Fields])) OR "deep learning"[All Fields]) OR ((("neural networks, computer"[MeSH Terms] OR (("neural"[All Fields] AND "networks"[All Fields]) AND "computer"[All Fields])) OR "computer neural networks"[All Fields]) OR ("neural"[All Fields] AND "network"[All Fields])) OR "neural network"[All Fields]) AND (("radiology"[MeSH Terms] OR "radiology"[All Fields] OR "radiography"[MeSH Terms] OR "radiography"[All Fields] OR "radiology s"[All Fields]) OR ("image"[All Fields] OR "image s"[All Fields] OR "imaged"[All Fields] OR "imager "[All Fields] OR "imagers"[All Fields] OR "images"[All Fields] OR "imaging"[All Fields] OR "imaging s"[All Fields] OR "imagings"[All Fields]) OR (("magnetic resonance imaging"[MeSH Terms] OR (("magnetic"[All Fields] AND "resonance"[All Fields]) AND "imaging"[All Fields])) OR "magnetic resonance imaging"[All Fields]) OR (((("tomography, x-ray computed"[MeSH Terms] OR (("tomography"[All Fields] AND "x ray"[All Fields]) AND "computed"[All Fields])) OR "x-ray computed tomography"[All Fields]) OR ("computed"[All Fields] AND "tomography"[All Fields])) OR "computed tomography"[All Fields]) OR ((((((((("diagnostic imaging"[MeSH Subheading] OR ("diagnostic"[All Fields] AND "imaging"[All Fields])) OR "diagnostic imaging"[All Fields]) OR "ultrasound"[All Fields]) OR "ultrasonography"[MeSH Terms]) OR "ultrasonography"[All Fields]) OR "ultrasonics"[MeSH Terms]) OR "ultrasonics"[All Fields]) OR "ultrasounds"[All Fields]) OR "ultrasound s"[All Fields]) OR ("radiomic"[All Fields] OR "radiomics"[All Fields]) OR ("tomosynthesis"[All Fields])) AND ((("chested"[All Fields] OR "thorax"[MeSH Terms] OR "thorax"[All Fields] OR "chest"[All Fields] OR "chests"[All Fields]) OR ("respiratory"[All Fields]) OR ("lung"[MeSH Terms] OR "lung"[All Fields]) AND ("pneumonia"[MeSH Terms] OR "pneumonia"[All Fields] OR "pneumoniae"[All Fields] OR "pneumonias"[All Fields] OR "pneumoniae s"[All Fields]) OR ((((((("covid 19"[All Fields] OR "covid 2019"[All Fields]) OR "severe acute respiratory syndrome coronavirus 2"[Supplementary Concept]) OR "severe acute respiratory syndrome coronavirus 2"[All Fields]) OR "2019 ncov"[All Fields]) OR "sars cov 2"[All Fields]) OR "2019ncov"[All Fields]) OR (("wuhan"[All Fields] AND ("coronavirus"[MeSH Terms] OR "coronavirus"[All Fields])))))

**Scopus Search string:**

(TITLE-ABS-KEY(artificial W/2 intelligence) OR TITLE-ABS-KEY(machine W/2 intelligence) OR TITLE-ABS-KEY(deep W/2 learning) OR TITLE-ABS-KEY(hierarchical W/2 learning) OR TITLE-ABS-KEY(artificial W/2 neural W/2 network) OR TITLE-ABS-KEY(algorithmic W/2 neural W/2 network) OR TITLE-ABS-KEY(computational W/2 neural W/2 network) OR TITLE-ABS-KEY(computer W/2 neural W/2 network) OR TITLE-ABS-KEY(computerized W/2 neural W/2 network) OR TITLE-ABS-KEY(connectionist W/2 model) OR TITLE-ABS-KEY(connectionist W/2 network) OR TITLE-ABS-KEY(connectionist W/2 neural W/2 network) OR TITLE-ABS-KEY(mathematical W/2 neural W/2 network) OR TITLE-ABS-KEY(neural W/2 network W/2 algorithm) OR TITLE-ABS-KEY(neural W/2 network W/2 model) OR TITLE-ABS-KEY(machine W/2 learning)) AND (TITLE-ABS-KEY(imaging) OR TITLE-ABS-KEY(radiomics) OR TITLE-ABS-KEY(nuclear W/2 magnetic W/2 resonance W/2 imaging) OR TITLE-ABS-KEY(mri) OR TITLE-ABS-KEY(nmr W/2 imaging) OR TITLE-ABS-KEY(imaging W/2 magnetization W/2 transfer) OR TITLE-ABS-KEY(magnetic W/2 resonance W/2 imaging) OR TITLE-ABS-KEY(magnetic W/2 resonance W/2 tomography) OR TITLE-ABS-KEY(mr W/2 imaging) OR TITLE-ABS-KEY(ultrasound) OR TITLE-ABS-KEY(phonophoresis) OR TITLE-ABS-KEY(radiation W/2 ultrasonic) OR TITLE-ABS-KEY(sonication) OR TITLE-ABS-KEY(sonification) OR TITLE-ABS-KEY(ultra sound) OR TITLE-ABS-KEY(ultrashell) OR TITLE-ABS-KEY(ultrasonic) OR TITLE-ABS-KEY(ultrasonic W/2 energy) OR TITLE-ABS-KEY(ultrasonic W/2 irradiation) OR TITLE-ABS-KEY(ultrasonic W/2 measurement) OR TITLE-ABS-KEY(ultrasonic W/2 sound) OR TITLE-ABS-KEY(ultrasonic W/2 wave) OR TITLE-ABS-KEY(ultrasound W/2 radiation) OR TITLE-ABS-KEY(radiology) OR TITLE-ABS-KEY(radiologic W/2 investigation) OR TITLE-ABS-KEY(radiologic W/2 technology) OR TITLE-ABS-KEY(radiological W/2 investigation) OR TITLE-ABS-KEY(radiological W/2 technology) OR TITLE-ABS-KEY(roentgenologic W/2 investigation) OR TITLE-ABS-KEY(roentgenological W/2 investigation) OR TITLE-ABS-KEY(roentgenology) OR TITLE-ABS-KEY(rontgenology) OR TITLE-ABS-KEY(tomosynthesis) OR TITLE-ABS-KEY(x W/1 ray W/2 analysis) OR TITLE-ABS-KEY(roentgen W/2 analysis) OR TITLE-ABS-KEY(roentgen W/2 ray W/2 analysis) OR TITLE-ABS-KEY(computer W/2 assisted W/2 tomography) OR TITLE-ABS-KEY(cat W/2 scan) OR TITLE-ABS-KEY(cat W/2 scanning) OR TITLE-ABS-KEY(computed W/2 tomographic W/2 scan) OR TITLE-ABS-KEY(computed W/2 tomography) OR TITLE-ABS-KEY(computed W/2 tomography W/2 scan) OR TITLE-ABS-KEY(computer W/2 tomography) OR TITLE-ABS-KEY(computerised W/2 axial W/2 tomography) OR TITLE-ABS-KEY(computerised W/2 tomography) OR TITLE-ABS-KEY(computerized tomography W/2 scan)) AND (TITLE-ABS-KEY(lung) OR TITLE-ABS-KEY(pulmo) OR TITLE-ABS-KEY(bronchus) OR TITLE-ABS-KEY(bronchial W/2 arch) OR TITLE-ABS-KEY(bronchial W/2 system) OR TITLE-ABS-KEY(bronchial W/2 tract) OR TITLE-ABS-KEY(thorax) OR TITLE-ABS-KEY(chest) OR TITLE-ABS-KEY(thoracic W/2 index) OR TITLE-ABS-KEY(thoracic W/2 region) OR TITLE-ABS-KEY(respiratory W/2 system) OR TITLE-ABS-KEY(apparatus W/2 respiratorius) OR TITLE-ABS-KEY(respiration W/2 system) OR TITLE-ABS-KEY(respiration W/2 tract) OR TITLE-ABS-KEY(respiratory W/2 apparatus) OR TITLE-ABS-KEY(respiratory W/2 track) OR TITLE-ABS-KEY(respiratory W/2 tract) OR TITLE-ABS-KEY(systema W/2 respiratorium)) AND ( TITLE-ABS-KEY(covid 19) OR TITLE-ABS-KEY(pneumonia) OR TITLE-ABS-KEY(acute W/2 diffuse W/2 pneumonia) OR TITLE-ABS-KEY(atrophic W/2 reticular W/2 pneumonia) OR TITLE-ABS-KEY(enzootic W/2 pneumonia) OR TITLE-ABS-KEY(inflammation W/2 lung) OR TITLE-ABS-KEY(inflammatory W/2 lung W/2 disease) OR TITLE-ABS-KEY(lobitis) OR TITLE-ABS-KEY(nonspecific W/2 inflammatory W/2 lung W/2 disease) OR TITLE-ABS-KEY(peripneumonia) OR TITLE-ABS-KEY(pleurisy W/2 pneumonia) OR TITLE-ABS-KEY(pleurisy) OR TITLE-ABS-KEY(pleuritic W/2 pneumonia) OR TITLE-ABS-KEY(pleuritis W/2 pneumonia) OR TITLE-ABS-KEY(pleuropneumonia) OR TITLE-ABS-KEY(pleuropneumonitis) OR TITLE-ABS-KEY(pneumonia W/2 pleuritica) OR TITLE-ABS-KEY(pneumonia W/2 superficialis) OR TITLE-ABS-KEY(pneumonic W/2 lung) OR TITLE-ABS-KEY(pneumonic W/2 pleurisy) OR TITLE-ABS-KEY(pneumonic W/2 pleuristis) OR TITLE-ABS-KEY(pneumonitis) OR TITLE-ABS-KEY(pulmonal W/2 inflammation) OR TITLE-ABS-KEY(pulmonary W/2 inflammation) OR TITLE-ABS-KEY(pulmonic W/2 inflammation) OR TITLE-ABS-KEY(stable pneumonia) OR TITLE-ABS-KEY(superficial W/2 pneumonia) OR TITLE-ABS-KEY(respiratory W/2 tract W/2 disease) OR TITLE-ABS-KEY(airway W/2 disease) OR TITLE-ABS-KEY(airway W/2 disorder) OR TITLE-ABS-KEY(respiration W/2 disease) OR TITLE-ABS-KEY(respiration W/2 tract W/2 disease) OR TITLE-ABS-KEY(respiratory W/2 disease) OR TITLE-ABS-KEY(respiratory W/2 disorder) OR TITLE-ABS-KEY(respiratory W/2 illness) OR TITLE-ABS-KEY(respiratory W/2 tract W/2 disorder))

**Embase search string**

('artificial intelligence'/mj OR 'artificial intelligence' OR 'machine intelligence' OR 'deep learning'/mj OR 'deep learning' OR 'hierarchical learning' OR 'artificial neural network'/mj OR 'algorithmic neural network' OR 'artificial neural network' OR 'artificial neural networks' OR 'computational neural network' OR 'computer neural network' OR 'computerized neural network' OR 'connectionist model' OR 'connectionist network' OR 'connectionist neural network' OR 'mathematical neural network' OR 'neural network (artificial)' OR 'neural network (computer)' OR 'neural network algorithm' OR 'neural network model' OR 'neural networks (computer)' OR 'neural networks, computer' OR 'machine learning'/mj OR 'learning machine' OR 'learning machines' OR 'machine learning') AND ('imaging'/mj OR 'imaging' OR 'radiomics'/mj OR 'radiomics' OR 'nuclear magnetic resonance imaging'/mj OR 'mri' OR 'nmr imaging' OR 'imaging, magnetization transfer' OR 'magnetic resonance imaging' OR 'magnetic resonance tomography' OR 'magnetization transfer imaging' OR 'mr imaging' OR 'nuclear magnetic resonance imaging' OR 'ultrasound'/mj OR 'phonophoresis' OR 'radiation, ultrasonic' OR 'sonication' OR 'sonification' OR 'ultra sound' OR 'ultrashell' OR 'ultrasonic' OR 'ultrasonic energy' OR 'ultrasonic irradiation' OR 'ultrasonic measurement' OR 'ultrasonic sound' OR 'ultrasonic wave' OR 'ultrasonic waves' OR 'ultrasonics' OR 'ultrasound' OR 'ultrasound radiation' OR 'radiology'/mj OR 'radiologic investigation' OR 'radiologic technology' OR 'radiological investigation' OR 'radiological technology' OR 'radiology' OR 'roentgenologic investigation' OR 'roentgenological investigation' OR 'roentgenology' OR 'rontgenology' OR 'technology, radiologic' OR 'tomosynthesis'/mj OR 'x ray analysis'/mj OR 'roentgen analysis' OR 'roentgen ray analysis' OR 'x ray analysis' OR 'computer assisted tomography'/mj OR 'cat scan' OR 'cat scanning' OR 'computed tomographic scan' OR 'computed tomography' OR 'computed tomography scan' OR 'computer assisted tomography' OR 'computer tomography' OR 'computerised axial tomography' OR 'computerised tomography' OR 'computerized axial tomography' OR 'computerized tomography' OR 'computerized tomography scan') AND ('lung'/mj OR 'lung' OR 'pulmo' OR 'bronchus'/mj OR 'bronchi' OR 'bronchial arch' OR 'bronchial system' OR 'bronchial tract' OR 'bronchus' OR 'thorax'/mj OR 'chest' OR 'thoracic index' OR 'thoracic region' OR 'thorax' OR 'respiratory system'/mj OR 'apparatus respiratorius' OR 'respiration system' OR 'respiration tract' OR 'respiratory apparatus' OR 'respiratory system' OR 'respiratory track' OR 'respiratory tract' OR 'systema respiratorium') AND ('covid 19'/mj OR 'pneumonia'/mj OR 'acute diffuse pneumonia' OR 'atrophic reticular pneumonia' OR 'enzootic pneumonia' OR 'inflammation, lung' OR 'inflammatory lung disease' OR 'lobitis' OR 'lung inflammation' OR 'nonspecific inflammatory lung disease' OR 'peripneumonia' OR 'pleurisy, pneumonia' OR 'pleuritic pneumonia' OR 'pleuritis, pneumonia' OR 'pleuropneumonia' OR 'pleuropneumonitis' OR 'pneumonia' OR 'pneumonia pleuritica' OR 'pneumonia superficialis' OR 'pneumonic lung' OR 'pneumonic pleurisy' OR 'pneumonic pleuritis' OR 'pneumonitis' OR 'pulmonal inflammation' OR 'pulmonary inflammation' OR 'pulmonic inflammation' OR 'stable pneumonia' OR 'superficial pneumonia' OR 'respiratory tract disease'/mj OR 'airway disease' OR 'airway disorder' OR 'respiration disease' OR 'respiration tract disease' OR 'respiratory disease' OR 'respiratory disorder' OR 'respiratory illness' OR 'respiratory tract disease' OR 'respiratory tract diseases' OR 'respiratory tract disorder')

**Table 3: QUADAS 2 overview of domains, signalling questions and analysis**

**DOMAIN 1: PATIENT SELECTION**

1. Was a consecutive or random sample of patients enrolled?
2. Was a case-control design avoided?
3. Are the patients’ inclusion/exclusion criteria as well as the study population described appropriately?
4. Was there a detailed description of the study population?

**DOMAIN 2: INDEX TEST(S)**

1. Is the description of imaging acquisition/processing protocol and segmentation method (where relevant) detailed?
2. Were the index test results by the human reader interpreted without knowledge of the results of the reference standard?

**DOMAIN 3: REFERENCE STANDARD**

1. Is the reference standard described?

**DOMAIN 4: FLOW AND TIMING**

1. Was the interval between index test and reference standard described?

**DOMAIN 5: Data management**

1. Was there a clear description of the data source?

**QUADAS-2 analysis**

In our QUADAS-2 assessment, we found that for the domain of patient selection, 17% of studies had a high or unclear risk of bias related to a case-control design of studies and insufficient description of the study population. In the Index test domain, we saw an unclear or risk of bias in 17% of studies, related to lack of information whether index test results were interpreted without knowledge of the results of the reference standard. In the reference standard domain, 33% of studies reported high or unclear risk of bias related to lack or unclear description of the reference standard. In the domain of flow and timing, we saw a high or unclear risk of bias in 42% of studies related to lacking or unclear information regarding intervals between PCR and imaging. In the additional domain of data management, there was an unclear risk of bias in 8% of studies related to unclear description of data sources used. We excluded a total of eight studies due to a predominant high risk throughout most domains.

**Table 4: PRISMA-DTA C**hecklist

| **Section/topic** | **#** | **PRISMA-DTA Checklist Item** | **Reported on page #** |
| --- | --- | --- | --- |
| **TITLE / ABSTRACT** | | |  |
| Title | 1 | Identify the report as a systematic review (+/- meta-analysis) of diagnostic test accuracy (DTA) studies. | Separate upload |
| Abstract | 2 | Abstract: See PRISMA-DTA for abstracts. | p. 1 |
| **INTRODUCTION** | | |  |
| Rationale | 3 | Describe the rationale for the review in the context of what is already known. | pp.2-3 |
| Clinical role of index test | D1 | State the scientific and clinical background, including the intended use and clinical role of the index test, and if applicable, the rationale for minimally acceptable test accuracy (or minimum difference in accuracy for comparative design). | pp.2-5 |
| Objectives | 4 | Provide an explicit statement of question(s) being addressed in terms of participants, index test(s), and target condition(s). | p.3-5 |
| **METHODS** | | |  |
| Protocol and registration | 5 | Indicate if a review protocol exists, if and where it can be accessed (e.g., Web address), and, if available, provide registration information including registration number. | Annex Tab. 1 |
| Eligibility criteria | 6 | Specify study characteristics (participants, setting, index test(s), reference standard(s), target condition(s), and study design) and report characteristics (e.g., years considered, language, publication status) used as criteria for eligibility, giving rationale. | pp. 4-5 and Annex Tab. 1 |
| Information sources | 7 | Describe all information sources (e.g., databases with dates of coverage, contact with study authors to identify additional studies) in the search and date last searched. | p. 5 |
| Search | 8 | Present full search strategies for all electronic databases and other sources searched, including any limits used, such that they could be repeated. | Annex Tab. 2 |
| Study selection | 9 | State the process for selecting studies (i.e., screening, eligibility, included in systematic review, and, if applicable, included in the meta-analysis). | pp. 5-6, fig 2 |
| Data collection process | 10 | Describe method of data extraction from reports (e.g., piloted forms, independently, in duplicate) and any processes for obtaining and confirming data from investigators. | pp.5-6 |
| Definitions for data extraction | 11 | Provide definitions used in data extraction and classifications of target condition(s), index test(s), reference standard(s) and other characteristics (e.g. study design, clinical setting). | pp.5-6, Tab. 1a and 1b |
| Risk of bias and applicability | 12 | Describe methods used for assessing risk of bias in individual studies and concerns regarding the applicability to the review question. | pp. 5-6, fig. 1a and1 b, Annex tab. 3 |
| Diagnostic accuracy measures | 13 | State the principal diagnostic accuracy measure(s) reported (e.g. sensitivity, specificity) and state the unit of assessment (e.g. per-patient, per-lesion). | p. 6 |
| Synthesis of results | 14 | Describe methods of handling data, combining results of studies and describing variability between studies. This could include, but is not limited to: a) handling of multiple definitions of target condition. b) handling of multiple thresholds of test positivity, c) handling multiple index test readers, d) handling of indeterminate test results, e) grouping and comparing tests, f) handling of different reference standards | p. 3 |

| **Section/topic** | **#** | **PRISMA-DTA Checklist Item** | **Reported on page #** |
| --- | --- | --- | --- |
| Meta-analysis | D2 | Report the statistical methods used for meta-analyses, if performed. | n.a. |
| Additional analyses | 16 | Describe methods of additional analyses (e.g., sensitivity or subgroup analyses, meta-regression), if done, indicating which were pre-specified. | n.a. |
| **RESULTS** | | |  |
| Study selection | 17 | Provide numbers of studies screened, assessed for eligibility, included in the review (and included in meta-analysis, if applicable) with reasons for exclusions at each stage, ideally with a flow diagram. | Fig. 2 |
| Study characteristics | 18 | For each included study provide citations and present key characteristics including: a) participant characteristics (presentation, prior testing), b) clinical setting, c) study design, d) target condition definition, e) index test, f) reference standard, g) sample size, h) funding sources | pp. 7-9, Tab. 1a and1b |
| Risk of bias and applicability | 19 | Present evaluation of risk of bias and concerns regarding applicability for each study. | fig. 1, supp. Info tab. 3 |
| Results of individual studies | 20 | For each analysis in each study (e.g. unique combination of index test, reference standard, and positivity threshold) report 2x2 data (TP, FP, FN, TN) with estimates of diagnostic accuracy and confidence intervals, ideally with a forest or receiver operator characteristic (ROC) plot. | Tab. 2, Tab 4, Figures 4 and 5 |
| Synthesis of results | 21 | Describe test accuracy, including variability; if meta-analysis was done, include results and confidence intervals. | pp.9-11 |
| Additional analysis | 23 | Give results of additional analyses, if done (e.g., sensitivity or subgroup analyses, meta-regression; analysis of index test: failure rates, proportion of inconclusive results, adverse events). | n.a. |
| **DISCUSSION** | | |  |
| Summary of evidence | 24 | Summarize the main findings including the strength of evidence. | pp.12-14 |
| Limitations | 25 | Discuss limitations from included studies (e.g. risk of bias and concerns regarding applicability) and from the review process (e.g. incomplete retrieval of identified research). | p. 14 |
| Conclusions | 26 | Provide a general interpretation of the results in the context of other evidence. Discuss implications for future research and clinical practice (e.g. the intended use and clinical role of the index test). | p.15 |
| **FUNDING** | | |  |
| Funding | 27 | For the systematic review, describe the sources of funding and other support and the role of the funders. | separate form |

*Adapted From:*  McInnes MDF, Moher D, Thombs BD, McGrath TA, Bossuyt PM, The PRISMA-DTA Group (2018). Preferred Reporting Items for a Systematic Review and Meta-analysis of Diagnostic Test Accuracy Studies: The PRISMA-DTA Statement. JAMA. 2018 Jan 23;319(4):388-396. doi: 10.1001/jama.2017.19163.For more information, visit: [**www.prisma-statement.org**](http://www.prisma-statement.org/)

**Table 5: Statistical analysis information**

**Key findings of the statistical analysis after verification of main performance measurements on all available datasets:**

- All studies have used more than one performance parameter, for example specificity and sensitivity (or recall), with 75% of these indicating corresponding 95% C.I. Over half of studies have compared these parameters, for example by using the McNemar test.
- PPV (or precision) has been used in 58% of the papers while NPV has been used in 42%. Their 95% C.I. have been reported in 17% of the papers.
- Two papers used the precision parameter. However, in one of them, the precision value is different from the PPV value.
- One study has used the AUC metric of the Precision Recall curve and all studies have used the AUC metric of the ROC curve; with 58% indicating corresponding 95% C.I. 42% of studies compared AUC values, for example by using the DeLong test.
- Half of the papers analysed the accuracy of the diagnostic tests, with half of these calculating 95% C.I.
- 33% of the papers computed the agreement (or concordance) between human readers and AI augmented readings. 25% of the papers analysed human readers supported by AI and 25% of the papers used the radiologist(s) consensus (or median) as reference standard in addition to RT-PCR.
- +LHR and –LHR, together with their 95% C.I., have been computed in only one paper.
- The Youden index has been mentioned in two papers without reporting corresponding numerical values.
- The F_1_ score is computed in 25% of the papers.
- Two papers computed the correlation (*i.e.*, linear correlation) between radiologists instead of focusing on agreement between radiologists and between radiologists and AI, as recommended by Gallagher and colleagues (1). 50% of the papers used the average (or median) of radiologists’ performance for the comparison with AI.
- The ICC has been used in these two papers. However, it characterises the IRR rather than the interested IRA, for example by using Cohen kappa index. Note that high ICC may be observed even though agreement is poor. Kappa statistic, which may be interpreted as the chance-corrected proportional agreement, is the best approach for assessing agreement between nonparametric dataset, as recommended by Gisev and colleagues (2).
- One paper focused on applying diagnostic test procedure on images knowing that several of them are associated to one patient.
- The training sample size has been mentioned in 3 papers. For optimising, one paper analyse the AUC metric of the AI system as polynomial fraction function of the training sample size.
- Power calculation was not reported, except for one paper that analysed the AUC as polynomial fraction function of the training sample size.

**Abbreviations**

AI artificial intelligence

AUC area under the curve

C.I. confidence interval

F_1_ harmonic mean of precision and recall

ICC intraclass correlation coefficient

IRA inter-rater agreement

IRR inter-rater reliability

+LHR positive likelihood ratio

-LHR negative likelihood ratio

PPV positive predictive value

NPV negative predictive value

ROC receiver operating characteristic

RT-PCR reverse transcription polymerase chain reaction

**Additional information:
List of full text articles assessed for eligibility: excluded papers with reasons**

1. **QUADAS-2 excluded due to high risk of bias (n=8)**

*(References and bibliography in full paper)*

Dorr et al.^3^

Javor et al.^4^

Ni et al.^5^

Wang Zheng et al.^6^

Yang Shuyi et al.^7^

Xie et al.^8^

Krishnamoorthy et al.^9^

Sukhija et al.^10^

1. **Full text articles excluded due to incomplete comparison/ no relevant results (n =14):**

Barbosa et al.^11^: incomplete comparison/ no relevant results

Liu et al.^12^: incomplete comparison/ no relevant results

Rangarajan et al.^13^: incomplete comparison/ no relevant results

Wang Lu et al.^14^: incomplete comparison/ no relevant results

Chen et al.^15^: incomplete comparison/ no relevant results

Ardakani et al.^16^: no comparison‚ performance of the proposed model was not compared with radiologists

Hwang et al.^17^: only AI supported reading, no other comparison

Jang et al.^18^: only COVID-19 with and without pneumonia, no comparison to patients without COVID-19

Jin et al.^19^: no relevant results reporting for human readers

Xu et al.^20^: incomplete comparison/ no relevant results (and not peer reviewed as of August 2021)

Blain et al.^21^: incomplete comparison/ no relevant results

Pu et al.^22^: incomplete comparison/ no relevant results

Tsiknakis et al.^23^: incomplete comparison/ no relevant results

Kumar et al.^24^: incomplete comparison/ no relevant results

**References**

1. Lessmann N, Sánchez CI, Beenen L, Boulogne LH, Brink M, Calli E, et al. Automated Assessment of COVID-19 Reporting and Data System and Chest CT Severity  Scores in Patients Suspected of Having COVID-19 Using Artificial Intelligence. Radiology. 2021 Jan;298(1):E18–28.

2. Zhang R, Tie X, Qi Z, Bevins NB, Zhang C, Griner D, et al. Diagnosis of Coronavirus Disease 2019 Pneumonia by Using Chest Radiography: Value of  Artificial Intelligence. Radiology. 2021 Feb;298(2):E88–97.

3. Wang M, Xia C, Huang L, Xu S, Qin C, Liu J, et al. Deep learning-based triage and analysis of lesion burden for COVID-19: a  retrospective study with external validation. The Lancet Digital health. 2020 Oct;2(10):e506–15.

4. Bai HX, Wang R, Xiong Z, Hsieh B, Chang K, Halsey K, et al. Artificial Intelligence Augmentation of Radiologist Performance in Distinguishing COVID-19 from Pneumonia of Other Origin at Chest CT. Radiology [Internet]. 2020/04/27. 2020 Sep;296(3):E156–65. Available from: https://pubmed.ncbi.nlm.nih.gov/32339081

5. Mei X, Lee H-C, Diao K, Huang M, Lin B, Liu C, et al. Artificial intelligence–enabled rapid diagnosis of patients with COVID-19. Nature Medicine [Internet]. 2020;26(8):1224–8. Available from: https://doi.org/10.1038/s41591-020-0931-3

6. Murphy K, Smits H, Knoops AJG, Korst MBJM, Samson T, Scholten ET, et al. COVID-19 on Chest Radiographs: A Multireader Evaluation of an Artificial  Intelligence System. Radiology. 2020 Sep;296(3):E166–72.

7. Wehbe RM, Sheng J, Dutta S, Chai S, Dravid A, Barutcu S, et al. DeepCOVID-XR: An Artificial Intelligence Algorithm to Detect COVID-19 on Chest  Radiographs Trained and Tested on a Large U.S. Clinical Data Set. Radiology. 2021 Apr;299(1):E167–76.

8. Zhou M, Yang D, Chen Y, Xu Y, Xu J-F, Jie Z, et al. Deep learning for differentiating novel coronavirus pneumonia and influenza  pneumonia. Annals of translational medicine. 2021 Jan;9(2):111.

9. Castiglioni I, Ippolito D, Interlenghi M, Monti CB, Salvatore C, Schiaffino S, et al. Machine learning applied on chest x-ray can aid in the diagnosis of COVID-19: a  first experience from Lombardy, Italy. European radiology experimental. 2021 Feb;5(1):7.

10. Wang H, Wang L, Lee EH, Zheng J, Zhang W, Halabi S, et al. Decoding COVID-19 pneumonia: comparison of deep learning and radiomics CT image  signatures. European journal of nuclear medicine and molecular imaging. 2020 Oct;1–9.

11. Chiu WHK, Vardhanabhuti V, Poplavskiy D, Yu PLH, Du R, Yap AYH, et al. Detection of COVID-19 Using Deep Learning Algorithms on Chest Radiographs. Journal of thoracic imaging. 2020 Sep.

12. Yang Y, Lure FYM, Miao H, Zhang Z, Jaeger S, Liu J, et al. Using artificial intelligence to assist radiologists in distinguishing COVID-19 from  other pulmonary infections. Journal of X-ray science and technology. 2021;29(1):1–17.

13. Gallagher EJ. Correlation versus agreement: methods of measurement in medicine. Vol. 27, Annals of emergency medicine. United States; 1996. p. 236–8.

14. Gisev N, Bell JS, Chen TF. Interrater agreement and interrater reliability: Key concepts, approaches, and applications. Res Soc Adm Pharm [Internet]. 2013;9(3):330–8. Available from: https://www.sciencedirect.com/science/article/pii/S1551741112000642

15. Dorr F, Chaves H, Serra MM, Ramirez A, Costa ME, Seia J, et al. COVID-19 pneumonia accurately detected on chest radiographs with artificial intelligence. Intell Med [Internet]. 2020;3–4:100014. Available from: https://www.sciencedirect.com/science/article/pii/S2666521220300144

16. Javor D, Kaplan H, Kaplan A, Puchner SB, Krestan C, Baltzer P. Deep learning analysis provides accurate COVID-19 diagnosis on chest computed tomography. Eur J Radiol. 2020 Dec;133:109402.

17. Ni Q, Sun ZY, Qi L, Chen W, Yang Y, Wang L, et al. A deep learning approach to characterize 2019 coronavirus disease (COVID-19) pneumonia in chest CT images. Eur Radiol. 2020 Dec;30(12):6517–27.

18. Wang Z, Xiao Y, Li Y, Zhang J, Lu F, Hou M, et al. Automatically discriminating and localizing COVID-19 from community-acquired pneumonia on chest X-rays. Pattern Recognit. 2021 Feb;110:107613.

19. Yang S, Jiang L, Cao Z, Wang L, Cao J, Feng R, et al. Deep learning for detecting corona virus disease 2019 (COVID-19) on high-resolution computed tomography: a pilot study. Ann Transl Med. 2020 Apr;8(7):450.

20. Xie Q, Lu Y, Xie X, Mei N, Xiong Y, Li X, et al. The usage of deep neural network improves distinguishing COVID-19 from other suspected viral pneumonia by clinicians on chest CT: a real-world study. Eur Radiol. 2020 Dec;1–10.

21. Krishnamoorthy S, Ramakrishnan S, Colaco LB, Dias A, Gopi IK, Gowda GAG, et al. Comparing a deep learning model’s diagnostic performance to that of radiologists to detect Covid -19 features on chest radiographs. Indian J Radiol Imaging. 2021 Jan;31(Suppl 1):S53–60.

22. Sukhija A, Mahajan M, Joshi PC, Dsouza J, Seth NDN, Patil KH. Radiographic findings in COVID-19: Comparison between AI and radiologist. Indian J Radiol Imaging. 2021 Jan;31(Suppl 1):S87–93.

23. Mortani Barbosa EJJ, Gefter WB, Ghesu FC, Liu S, Mailhe B, Mansoor A, et al. Automated Detection and Quantification of COVID-19 Airspace Disease on Chest Radiographs: A Novel Approach Achieving Expert Radiologist-Level Performance Using a Deep Convolutional Neural Network Trained on Digital Reconstructed Radiographs From Computed. Invest Radiol. 2021 Aug;56(8):471–9.

24. Liu H, Ren H, Wu Z, Xu H, Zhang S, Li J, et al. CT radiomics facilitates more accurate diagnosis of COVID-19 pneumonia: compared with CO-RADS. J Transl Med. 2021 Jan;19(1):29.

25. Rangarajan K, Muku S, Garg AK, Gabra P, Shankar SH, Nischal N, et al. Artificial Intelligence-assisted chest X-ray assessment scheme for COVID-19. Eur Radiol. 2021 Aug;31(8):6039–48.

26. Wang L, Kelly B, Lee EH, Wang H, Zheng J, Zhang W, et al. Multi-classifier-based identification of COVID-19 from chest computed tomography using generalizable and interpretable radiomics features. Eur J Radiol. 2021 Mar;136:109552.

27. Chen J, Wu L, Zhang J, Zhang L, Gong D, Zhao Y, et al. Deep learning-based model for detecting 2019 novel coronavirus pneumonia on high-resolution computed tomography. Sci Rep. 2020 Nov;10(1):19196.

28. Ardakani AA, Kanafi AR, Acharya UR, Khadem N, Mohammadi A. Application of deep learning technique to manage COVID-19 in routine clinical practice using CT images: Results of 10 convolutional neural networks. Comput Biol Med. 2020 Jun;121:103795.

29. Hwang EJ, Kim H, Yoon SH, Goo JM, Park CM. Implementation of a Deep Learning-Based Computer-Aided Detection System for the Interpretation of Chest Radiographs in Patients Suspected for COVID-19. Korean J Radiol. 2020 Oct;21(10):1150–60.

30. Jang SB, Lee SH, Lee DE, Park S-Y, Kim JK, Cho JW, et al. Deep-learning algorithms for the interpretation of chest radiographs to aid in the triage of COVID-19 patients: A multicenter retrospective study. PLoS One. 2020;15(11):e0242759.

31. Jin C, Chen W, Cao Y, Xu Z, Tan Z, Zhang X, et al. Development and evaluation of an artificial intelligence system for COVID-19 diagnosis. Nat Commun. 2020 Oct;11(1):5088.

32. Xu Y, Ma L, Yang F, Chen Y, Ma K, Yang J, et al. A collaborative online AI engine for CT-based COVID-19 diagnosis. medRxiv : the preprint server for health sciences. 2020.

33. Blain M, Kassin MT, Varble N, Wang X, Xu Z, Xu D, et al. Determination of disease severity in COVID-19 patients using deep learning in chest X-ray images. Diagn Interv Radiol. 2021 Jan;27(1):20–7.

34. Pu J, Leader J, Bandos A, Shi J, Du P, Yu J, et al. Any unique image biomarkers associated with COVID-19? Eur Radiol. 2020 Nov;30(11):6221–7.

35. Tsiknakis N, Trivizakis E, Vassalou EE, Papadakis GZ, Spandidos DA, Tsatsakis A, et al. Interpretable artificial intelligence framework for COVID-19 screening on chest X-rays. Exp Ther Med. 2020 Aug;20(2):727–35.

36. Vijay Kumar J, Harshavardhan A, Bhukya H, Krishna Prasad A V. Advanced Machine Learning-Based Analytics on COVID-19 Data Using Generative Adversarial Networks. Mater today Proc. 2020 Oct;
